# Genomic reconstruction of fossil and living microorganisms in ancient Siberian permafrost

**DOI:** 10.1186/s40168-021-01057-2

**Published:** 2021-05-17

**Authors:** Renxing Liang, Zhou Li, Maggie C. Y. Lau Vetter, Tatiana A. Vishnivetskaya, Oksana G. Zanina, Karen G. Lloyd, Susan M. Pfiffner, Elizaveta M. Rivkina, Wei Wang, Jessica Wiggins, Jennifer Miller, Robert L. Hettich, Tullis C. Onstott

**Affiliations:** 1 Princeton University, B88, Guyot Hall, Princeton, NJ 08544 USA; 2 Biosciences Division, Oak Ridge National Laboratory, Oak Ridge, TN USA; 3 Present address: Institute of Deep-sea Science and Engineering, Chinese Academy of Sciences, Sanya, China; 4 University of Tennessee, Knoxville, TN USA; 5 Institute of Physicochemical and Biological Problems in Soil Science, Russian Academy of Sciences, Pushchino, Moscow Region Russia; 6 Genomics Core Facility, Princeton University, Princeton, NJ USA

**Keywords:** Ancient permafrost, Metagenome-assembled genome, Fossil and living microorganisms, Long-term survivability, Adaptive strategy, Asgard archaea

## Abstract

**Background:**

Total DNA (intracellular, iDNA and extracellular, eDNA) from ancient permafrost records the mixed genetic repository of the past and present microbial populations through geological time. Given the exceptional preservation of eDNA under perennial frozen conditions, typical metagenomic sequencing of total DNA precludes the discrimination between fossil and living microorganisms in ancient cryogenic environments. DNA repair protocols were combined with high throughput sequencing (HTS) of separate iDNA and eDNA fraction to reconstruct metagenome-assembled genomes (MAGs) from ancient microbial DNA entrapped in Siberian coastal permafrost.

**Results:**

Despite the severe DNA damage in ancient permafrost, the coupling of DNA repair and HTS resulted in a total of 52 MAGs from sediments across a chronosequence (26–120 kyr). These MAGs were compared with those derived from the same samples but without utilizing DNA repair protocols. The MAGs from the youngest stratum showed minimal DNA damage and thus likely originated from viable, active microbial species. Many MAGs from the older and deeper sediment appear related to past aerobic microbial populations that had died upon freezing. MAGs from anaerobic lineages, including *Asgard* archaea, however exhibited minimal DNA damage and likely represent extant living microorganisms that have become adapted to the cryogenic and anoxic environments. The integration of aspartic acid racemization modeling and metaproteomics further constrained the metabolic status of the living microbial populations. Collectively, combining DNA repair protocols with HTS unveiled the adaptive strategies of microbes to long-term survivability in ancient permafrost.

**Conclusions:**

Our results indicated that coupling of DNA repair protocols with simultaneous sequencing of iDNA and eDNA fractions enabled the assembly of MAGs from past and living microorganisms in ancient permafrost. The genomic reconstruction from the past and extant microbial populations expanded our understanding about the microbial successions and biogeochemical alterations from the past paleoenvironment to the present-day frozen state. Furthermore, we provided genomic insights into long-term survival mechanisms of microorganisms under cryogenic conditions through geological time. The combined strategies in this study can be extrapolated to examine other ancient non-permafrost environments and constrain the search for past and extant extraterrestrial life in permafrost and ice deposits on Mars.

**Video abstract**

**Supplementary Information:**

The online version contains supplementary material available at 10.1186/s40168-021-01057-2.

## Introduction

Permafrost underlies 25% of the Northern Hemisphere and 20% of Earth*’*s land surface [1]. Depending on the geographic location, heat flow, and host deposit type, permafrost can extend several hundred meters into the Earth’s surface [2]. Permafrost is normally defined as subsurface material that remains continuously frozen for at least 2 years, underlying an annually thawed active layer. The near-surface layers of permafrost typically are Holocene in age, whereas much older and deeper permafrost deposits in Siberia, Yukon valley (Canada), and Antarctica have been reported to be continuously frozen since their formation in the Pleistocene and even Pliocene epochs [3–7]. In Pliocene/Pleistocene sedimentary permafrost deposits, the post-depositional cooling and freezing should have a drastic impact on the entrapped community of all living organisms. Most eukaryotes (fauna and flora) and many prokaryotes likely died and became fossilized. However, certain microorganisms may have adapted to the subzero temperatures and have remained metabolically active in the frozen sediments for thousands to millions of years [1, 3–5, 8]. Therefore, the ancient permafrost sediments represent an archive of both dead microorganisms (molecular fossil remains) and presently living microbial populations that have adapted to the cryogenic environment over geological time.

Most previous cryopreservation studies have centered on the paleoecology and paleogenomics of mammals, plants, and fungi by sequencing the relic DNA recovered from fossil remains in ancient permafrost and cave deposits [9–11]. Although the microbial life in permafrost has been studied by culture-dependent and DNA-based molecular techniques for many decades [1, 12], the demarcation between fossil and living microorganisms, particularly in ancient frozen sediment, remains a major underexplored question in the microbial ecology of permafrost. Various traditional approaches such as cultivation [13], stable isotope probing [14], depletion of DNA from dead cells [15], and Live/Dead microscopic assays [8, 15–18] have been employed to identify metabolically active microorganisms in ancient permafrost. However, none of these approaches have provided insights into the paleoecology and paleogenomics of dead microbial populations by targeting the relic DNA (molecular fossil remains) preserved under subzero temperatures through geological time. The total DNA pool in a given environment typically includes extracellular DNA (eDNA) largely liberated from dead cells, and intracellular DNA (iDNA) from living, dormant, and/or dead, but structurally intact cells [19, 20]. The exceptional preservation of eDNA and iDNA under perennial frozen conditions poses challenges for discriminating fossil and living organisms because most sequencing-based studies target the total DNA, which represents a mixed genetic repository of the past and viable microbial populations. A few recent studies have sequenced 16S ribosomal RNA (rRNA) gene amplicons from iDNA and eDNA fractions extracted from permafrost sediment [8] and from non-permafrost sediment of lacustrine-alluvial [21] or marine origins [22, 23]. The microbial taxa exclusively identified in the eDNA fraction have been deemed to represent past microbial communities that are no longer part of the living microbial community [8, 21]. Microbial taxa identified in both iDNA and eDNA fractions could either represent just fossil microorganisms present in intact dead cells as well as extracellular remains or they could represent living microorganisms whose ancestors were present in the fossil microbial community. The paleoecological status of those microbial taxa present in both the iDNA and eDNA fractions is therefore ambiguous. Moreover, genomic insight into the fossil versus living microorganisms in ancient permafrost is lacking because previous studies were based on 16S rRNA gene amplicon sequencing, which only provides putative taxonomic identity rather than genomic evidence of metabolic and ecological capabilities [8, 22–24].

Despite the exceptional preservation of DNA under frozen conditions, eDNA released from lysed cells and iDNA enclosed in structurally intact dead cells undergoes various types of DNA damage in ancient permafrost over geological time [25]. By contrast, the integrity of genomic DNA from metabolically active cells in ancient permafrost can be maintained by active cellular DNA repair [26, 27]. Therefore, together with the simultaneous separation of iDNA and eDNA fractions, the accumulated damage in the genomic DNA provides an additional line of evidence in discriminating between fossil and living microorganisms in ancient permafrost. Due to the severe damage in ancient fossil remains, laboratory DNA repair protocols have been frequently applied to facilitate sequencing of genomes of various eukaryotic organisms from ancient DNA in the field of paleontology. Double-stranded DNA that has degraded to single-stranded DNA and nucleotides that have been dimerized or deaminated have been repaired by a mixture of endonucleases and glycosylases that mimic in vivo DNA repair complexes [28]. However, the recovery of paleogenomes of fossil prokaryotes and even genomes of presently living microorganisms from ancient permafrost have been challenging due to the extremely low biomass [8, 18]. Moreover, the severe DNA damage and inherent chemical inhibitors associated with ancient permafrost can further preclude high DNA yield and efficient PCR amplification for sequencing [8, 18, 29]. In principle, DNA repair protocols should dramatically improve the genome recovery of fossil microbial species by enabling the sequencing of damaged DNA fragments that previously could not be sequenced. Therefore, we hypothesized that the integration of DNA repair protocols with metagenomic sequencing of iDNA and eDNA fractions would enable reconstruction of metagenome-assembled genomes (MAGs) from fossil microorganisms and enable identification of those taxa comprising fossil versus extant microorganisms.

Here, we coupled the PreCR DNA repair protocol [28] and genome-resolved metagenomics to interrogate ancient microbial communities preserved in an Arctic Siberian permafrost chronosequence that captures a transition from late Pleistocene (26–43 kyr freezing ages) fluvial fresh water sediments to middle Pleistocene (100–120 kyr freezing ages) marine sediments. By sequencing individual fractions of iDNA and eDNA separately with and without DNA repair, high-quality MAGs were successfully recovered from dead and contemporarily living microbial communities in ancient marine sediments frozen over 100 kyr ago. Moreover, aspartic acid racemization modeling and metaproteomics were integrated to constrain the metabolic status of the living microbial populations. Collectively, we provided a solution to discriminate the fossilized from the living microorganisms at genomic scale from ancient frozen sediment. The developed strategies for studying paleogenomics of microorganisms can be extrapolated to other ancient environments where relic DNA from past microbial communities might persist.

## Methods

### Sample collection and geochemical characterization

The sampling site is a continuous permafrost area along the coastline of the East Siberian Sea (Fig. S1) The mean annual temperature ranges from − 9 to − 11 °C and the thickness of the permafrost can be up to 800 m [2]. The stratification of the permafrost in this area is unique because a marine horizon (~ 20 m thick; at 4–24 m below the land surface) is sandwiched between non-saline terrigenous sediments (Fig. S2). This marine horizon, the Kon'kovaya suite, can be dated back to the end of the Middle Pleistocene [30]. The Kon'kovaya suite contains finely dispersed sand and sandy loams that were deposited and accumulated in the bottom of littoral lagoons at around 0 °C during a marine transgression [31]. During the subsequent regression of the polar ocean between 100 and 120 kyr ago, the Kon'kovaya suite was subaerially exposed for thousands of years and became frozen over time. The frozen marine horizon was covered between 26 and 43 kyr ago by a layer of lacustrine-alluvial sediment containing polygonal ice wedges forming what is referred to as the Late Pleistocene Ice Complex of the Yedoma suite (Fig. S2) [23]. Mid-Holocene sediments were deposited on the top of icy complex around 5–8 kyr ago and subsequently frozen (Fig. S2). The presence of polygonal ice wedges in the icy complex and their absence in the overlying Holocene sediments indicate that the icy complex and the underlying marine strata have never been thawed subsequent to freezing.

To collect permafrost sediment representing all layers of various geological ages, a 22-m-long vertical core, Ch1-17, was collected at Cape Chukochii near the East Siberian Sea coast (70° 05′ N, 159° 55′ E; Fig. S1) using the same drilling protocols and aseptic techniques as previously described [32, 33]. The intact core was sectioned at various intervals in the field and transported frozen to Princeton University on dry ice and stored at − 80 °C until analyses. Since the goal of the study was to determine if paleoecological insights could be recovered from ancient DNA, samples from three depth intervals (3.4, 5.8, and 14.8 m, meters below land surface, Fig. S2) were selected to sample the distinct paleoenvironments of the terrigenous to marine sediment transition as well as the span of geological ages (Fig. S2). The in situ temperature was determined at the time of sampling with an Onset® HOBO® Data Logger and pH was measured with a pH meter (Toledo, Seven Easy pH -meter). The anions and organic acids from the permafrost sediments were determined by ion chromatography (Dionex, CA, USA) as previously described [8].

### Determination of aspartic acid (Asp) racemization in bulk sediment and cellular proteins

The racemization of l-Asp has been used as an index to constrain microbial anabolic activity in various ancient samples including marine and permafrost sediments [8, 34]. A portion of the sediment (0.1 g) at each depth and cells separated from three selected depths (3.4, 5.8, and 14.8 m, Fig. S2) was used to quantify d- and l-Asp using high-performance liquid chromatography (HPLC) according to a previously established procedure [8]. The cells were separated from sediment materials (3 g) using multiple density gradient of Nycodenz and sodium polytungstate [35] with some modifications and the viability of cells was assessed visually using the LIVE/DEAD® BacLight Bacterial Viability kit (Invitrogen, Carlsbad, CA) as described elsewhere [8]. The sediment portion was hydrolyzed with 1 mL 6 N HCl at 105 °C for 16 h under N_2_. The hydrolysate (50 μL) was dried in a speed vacuum concentrator and the residues were resuspended with 1 mL Milli-Q H_2_O before analysis. The hydrolysis of separated cells was performed using the same protocol except that 0.5 mL 6 N HCl was used for the reaction. All samples were derivatized with o-phthaldialdehyde/*N*-acetyl-l-cysteine as previously described [36] prior to HPLC analysis. The instrument configurations and detailed procedures for operating HPLC has been reported in a previous study [8]. According to kinetic parameters of Asp racemization in ancient permafrost sediment from Siberia [37], the racemization rate at in situ temperature was calculated according to the Arrhenius equation:

1 $$ k=A{e}^{\left(\frac{-{E}_a}{RT}\right)} $$

in which *k* is the racemization rate constant (year^−1^), *E*_*a*_ is the activation energy (101.7 kJ mol^−1^), *A* is the frequency factor (1.43 × 10^15^ year^−1^), *R* refers to the universal gas constant (8.314 × 10^−3^ kJK^−1^ mol^−1^), and *T* is temperature in K [37]. Since the geological age at each layer (Fig. S2) was well documented [2, 31] and the temperature of the frozen sediment (Fig. S3) has been stable over geological time, the ratio of d/l Asp values can be predicted for each depth using the Eq. 2 below:

2 $$ {\mathit{\ln}}_t\left[\frac{1+D/L}{1-D/L}\right]-{\mathit{\ln}}_0\left[\frac{1+D/L}{1-D/L}\right]=2 kt $$

where d/l refers to the ratio of d-Asp to l-Asp and *t* is time.

### Extraction of intracellular and extracellular DNA

Permafrost sediments from three depths (3.4, 5.8, and 14.8 m) were used for simultaneous extraction of extracellular DNA (eDNA) and intracellular DNA (iDNA) using a modified protocol described in our earlier study [8]. Briefly, permafrost sediment (10 g) was mixed with a sterile phosphate buffer (0.12 M Na_2_HPO_4_ [pH 8]) and then centrifuged at 10,000×*g* for 10 min at 4 °C in order to separate the eDNA fraction into the aqueous phase. The remaining sediment was used to extract iDNA pool using DNeasy PowerMax soil kit (QIAGEN, Carlsbad, CA) according to the manufacturer’s procedures. The eDNA fraction in the supernatant was extracted using the standard procedures of the same kit except that the steps for bead beating and cell lysis were bypassed. A parallel extraction with sediment-free blank control was accompanied to monitor potential contamination introduced from the reagents and laboratory environment during extraction. The concentration of DNA was quantified using a Qubit 3.0 fluorometer with the dsDNA HS assay kit (Invitrogen, Carlsbad, CA, USA). Furthermore, the quality and size distribution of the iDNA and eDNA fractions were determined using Bioanalyzer DNA High Sensitivity chips (Agilent, CA). The DNA yield in the blank control was below detection (< 0.01 ng/μL) and was not included for metagenomic sequencing.

### DNA repair and metagenomic sequencing

Given the anticipated DNA damage in the ancient permafrost samples, both iDNA and eDNA fractions were subject to DNA repair with PreCR™ Repair Mix (New England Biolabs, MA, USA) prior to sequencing. The PreCR™ Repair Mix is an enzyme cocktail (containing *Taq* DNA Ligase, Endonuclease IV, *Bst* DNA Polymerase, *Fpg,* Uracil-DNA Glycosylase, T4 Endonuclease V, and Endonuclease VIII) that can repair a wide range of DNA damages such as deaminated cytosine, apurinic/apyrimidinic sites, thymine dimers, nicks, and gaps. Up to 50 ng of DNA was treated with PreCR™ Repair mix at 37 °C for 20 min according to the manufacturer’s instructions. The original untreated DNA, as well as the PreCR repaired DNA, were then converted to Illumina sequencing libraries using the Nextera DNA Flex Library Prep kit (Illumina, CA) with a unique DNA barcode added to each library. The libraries were examined on Bioanalyzer DNA High Sensitivity chips (Agilent, CA) for size distribution, and quantified by Qubit fluorometer (Invitrogen, CA). Each set of libraries were pooled at equal molar amount and sequenced on Illumina HiSeq 2500 Rapid flow cell as 2 × 150 nt paired-end reads. In total, 12 metagenomic libraries from the iDNA and eDNA fractions with and without DNA repair were sequenced. The pass-filter reads were retained and demultiplexed using fastq-multx for further analysis.

### Metagenomic assembly, binning, and annotation

The raw sequences were quality-filtered to remove Illumina sequencing adaptors and low-quality sequences using fastp v.0.12.6 [38] with the parameter (length < 50 nt and Phred scores < 30). The clean reads from all four metagenomes (iDNA, eDNA, iDNA_PreCR, and eDNA_PreCR) at each depth were co-assembled with MEGAHIT v1.1.4 [39] using paired-end mode with the settings of *k*-min = 27, *k*-max = 137, *k-*step = 10. The co-assembled contigs (> 1.5 kb) were binned using the default settings in the “Binning module” implemented in MetaWRAP v0.8 [40] that adopts three different tools, namely MetaBAT v2.12.1 [41], MaxBin v2.0 [42], and CONCOCT v1.1.0 [43]. The generated MAGs were refined with the “Bin_refinement module” in MetaWRAP v0.8 [40]. The refined MAGs were further re-assembled with the “Reassemble_bins module” in MetaWRAP v0.8 [40]. The quality of the reassembled MAGs were assessed with CheckM v1.0.11 [44]. The functional genes (protein coding sequences, CDS) from all MAGs were predicted and annotated using Prokka v1.13 [45] and DFAST tools [46] against TIGRFAM and COG databases. Furthermore, all CDS from each MAG were identified by blastp against NCBI nr database to obtain the top 10 hits for further confirmation. The metabolic pathways related to carbon, sulfur, and nitrogen metabolism were predicted using the automated annotation server RAST (Rapid Annotation using Subsystem Technology) with the default settings [47]. The carbohydrate active enzymes in the MAGs were annotated based on the HMMER (E-Value < 1e-15) tool implemented in the online sever dbCAN2 [48].

### Phylogeny and other genome-centric analyses

The taxonomic classification of all MAGs was determined using Genome Taxonomy Database Toolkit (GTDB-Tk v 0.3.0) [49] based on 120 bacterial and 122 archaeal marker genes. Closely related MAGs or genomes of cultivated organisms were downloaded from NCBI database (accessed in April, 2020) for phylogenomic analysis. The sequences of 16 single-copy ribosomal proteins were extracted and aligned with MUSCLE v3.8.31 in Anvi’o v5. 2[50]. The alignment was concatenated for building phylogenetic tree with RAxML v8.1.17 [51] using the PROTGAMMAILGF model for amino acid sequence evolution and 1000 bootstraps. The finalized phylogenetic tree was visualized using the online iTOL tool [52]. The in situ growth rates of microbial populations in the permafrost were inferred based on the Growth Rate Index (GRiD) derived from all bacterial MAGs [53]. The GRiD values were determined by mapping the metagenomic reads to each MAG in order to calculate the ratio of coverage at the peak (origin of replication, *ori*) and trough (terminus, *ter*) regions [53]. Meanwhile, the GRiD values were further constrained according to the coverage information of chromosome initiator replication gene (*dnaA*) and deletion-induced filamentation (*dif*) sequences across the genome [53]. The GRiD values were considered as invalid if *dnaA/ori* and *ter/dif* coverage ratios were above 0.8 and the species heterogeneity was below 0.3.

### Assessing genomic DNA damage

The damage to the genomic DNA was evaluated based on the impact of DNA repair on the completeness of MAGs and frequency of cytosine deamination in the iDNA and eDNA fraction. Briefly, the reads from four metagenomes (iDNA, eDNA, iDNA_PreCR, and eDNA_PreCR) at each depth (3.4, 5.8, and 14.8 m) were individually mapped to the each MAG reconstructed from the abovementioned co-assembly with BWA v0.7.15 implemented in MetaWRAP v0.8 using the “strict” option (no mismatches) [40]. The mapped reads from each MAG were reassembled using SPAdes v3.13.0 [54] and a set of k-mer sizes (21, 33, 55, 77) in MetaWRAP v0.8 [40]. The quality of the reassembled MAGs from each individual metagenome were assessed with CheckM v1.0.11 in order to compare the completeness of MAGs with and without DNA repair in metagenomes derived from both the iDNA and eDNA.

The nucleotide mis-incorporation pattern caused by cytosine deamination has been frequently used to assess the severity of damage in ancient DNA in historical samples [55]. As Uracil-DNA glycosylase removes uracil and insert cytosine back during the DNA repair process prior to sequencing, the frequency of cytosine deamination from the 5′-end of reads could be determined using mapDamage v2. 0[55]. Briefly, the MAGs derived from the iDNA metagenome after DNA repair was selected as reference genomes. Each reference genome was indexed with BWA v0.7.15 [56] and the reads from each metagenome were individually aligned to the indexed reference genome with the bwa aln algorithm and bwa samse [56]. The aligned sam files were converted to bam format and sorted using SAMtools [57]. The DNA damage pattern (C-T substitution) at the first 25 nucleotides from the 5′-end was estimated using mapDamage v2.0 [55] with the default parameters.

### Genome-resolved metaproteomic analyses

Due to the low biomass in ancient permafrost, 10 g of the sediment from three depths was used for protein extraction with NoviPure Soil Protein Extraction Kit (QIAGEN) with following modifications of the manufacture’s protocol, as described previously [58]. Cell lysates were concentrated using Amicon Ultra-4 Centrifugal Filter Units (30 kDa molecular weight cut-off; Millipore) to ~ 1 mL, and then proteins in the concentrated cell lysates were precipitated by trichloroacetic acid for overnight at 4 °C, pelleted by centrifugation at 4 °C, washed with ice-cold acetone three times, and re-solubilized in guanidine (6 M). Bicinchoninic acid assays were conducted to estimate the protein concentrations. Dithiothreitol (10 mM, final concentration) was added to reduce disulfide bonds. The filter-aided sample preparation method was used for further sample processing, as described previously [59]. Proteins were first trypsin-digested overnight in an enzyme-to-substrate ratio of 1:100 (weight:weight) with gentle shaking, followed by a second digestion for 4 h. All digested peptide samples were stored at − 80 °C. The peptides were analyzed with an 11-step online multidimensional protein identification technology [60] on an LTQ Orbitrap Elite mass spectrometer (Thermo Fisher Scientific) as described previously [58]. The acquired MS/MS data were searched using Sipros Ensemble [61] against the matched protein database constructed from the CDS from all MAGs. Initial results were filtered with a 1% FDR threshold at the peptide level estimated by the target–decoy approach [61]. The cutoff for protein identification was defined as one unique peptide which exclusively mapped to that identified protein. The relative abundance of protein expression from each MAG was based on the balanced spectral counts. The balanced spectral counts were achieved by summing the spectral counts uniquely mapping to a protein plus a fraction of the non-unique spectra split evenly between matching proteins [62].

## Results

### Geochemical characteristics

The in situ temperatures at various depths of the borehole varied from − 7 to − 8 °C and pH of the porewater was in the range of 6.5–7.4 (Additional file 1: Fig. S3). The major anions (Br^−^, Cl^−^, and SO_4_^2−^) generally increased with depth with an exception at 10.4 m (Additional file 1: Fig. S3). The concentrations of Cl^−^ and SO_4_^2−^ in the deeper sediments (13.9–18.3 m) were much higher (8783.2 ± 1831.2–9142 ± 1231 μg/g Cl^−^ and 851.6 ± 72.2–1047 ± 94.7 μg/g SO_4_^2−^) than those from the top layers (3.1–3.4 m) of the Yedoma suite (120.4 ± 17.3–144.7 ± 4.9 μg/g Cl^−^ and 7.2 ± 1.1–11.5 ± 0.9 μg/g SO_4_^2−^). Notably, the Cl^−^/Br^−^ ratios in the layers below 5.4 m (212.2–257.2) were much higher than those of the top layers (35.5–55.6). The higher salinity and the similarity of Cl^−^/Br^−^ in the deeper layers to that of seawater [63] (Cl^−^/Br^−^ ~ 294) confirmed that the Kon’kovaya suite (Additional file 1: Fig. S2) originated from marine sediments during the upper Middle Pleistocene transgression [31]. Low–molecular-weight organic acids (formate, acetate, and propionate) were detected at all depths with the highest concentration at 5.8 m (Additional file 1: Fig. S4). The concentration of acetate was in the range of 2.2 ± 0.39 to 36.2 ± 3.3 μg/g, whereas the content of formate varied from 1.0 ± 0.03 to 12.0 ± 0.7 μg/g.

### DNA damage and aspartic acid racemization

The DNA yield dramatically decreased with depth for both iDNA and eDNA fractions (Additional file 1: Fig. S5). The eDNA/iDNA ratios in the deeper, older layers at 5.8 and 14.8 m were 0.55 and 0.58, respectively, which are higher than the 0.3 value from the shallower, younger layer at 3.4 m, indicating that more relic DNA was liberated from dead cells or fewer cells remained intact in the deeper, older sediments. The size distribution of DNA fragments further confirmed that the eDNA fractions were more fragmented than the corresponding iDNA fractions of each sample (Additional file 1: Fig. S6). Moreover, the both iDNA and eDNA fractions from the marine horizons at 5.8 and 14.8 m was much more fragmented with the predominance of short sized DNA fragments (~ 100–300 bp) relative to those of the youngest sample at 3.4 m (Additional file 1: Fig. S6). The preponderance of short DNA fragments in both iDNA and eDNA fractions in the deeper, older layers (Additional file 1: Fig. S6) suggested that relic eDNA and iDNA encased in structurally intact dead cells (Additional file 1: Fig. S7) were severely damaged through geological time. The large DNA fragments (> 10.38 kb) present at all three depths were indicative of high integrity DNA with less damage from potentially live cells. Indeed, Live/Dead cell staining revealed that both live cells and structurally intact dead cells were isolated from all three depths (Additional file 1: Fig. S7). Notably, the low abundance of high-integrity DNA from the iDNA fraction from deeper strata at 5.8 and 14.8 m (Fig. S6) also coincided with the presence of a low number of living microbial cells (Additional file 1: Fig. S7).

**Fig. 1:**
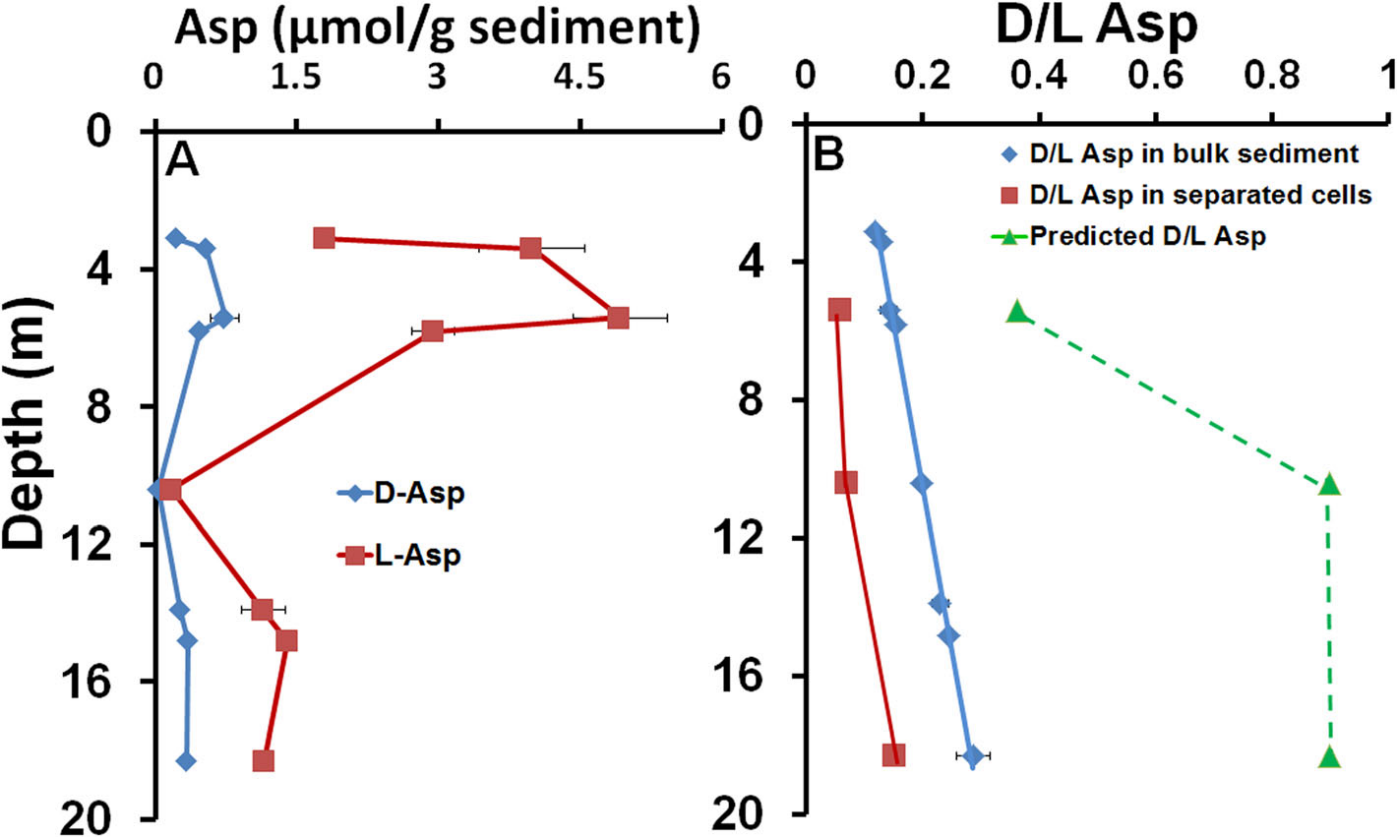
Concentration of d- and l-Aspartic acid in bulk sediment of the Middle Pleistocene marine Kon’kovaya suite (**a**); Measured d/l Asp in bulk sediment and separated cells (left) and the predicted d/l Asp calculated from Asp racemization rate, the geological ages of each depth of the permafrost (26 kyr for 3.4 m sample and 100 kyr for 5.8 and 14.8 samples for conservative estimation) and an assumed constant paleotemperature of − 7.7 °C. The equation (*y* = 94.213*x* - 8.2957) with high *R*^2^ (0.996) was deduced from the linear regression of the increasing d/l Asp with depth in the bulk sediment. The error bars represent the standard deviation from three biological replicates

Similar to the trend of DNA, the concentrations of d- and l-Asp generally decreased with depth (Fig. 1a), confirming the low biomass in the deeper sediment of greater geological age (Additional file 1: Fig. S5). The d/l Asp in the bulk sediment gradually increased from 0.12 to 0.29 with increasing depth from 3.4 to 18.3 m and followed a remarkably linear relationship (*R*^2^ = 0.996) (Fig. 1b). Although the increasing d/l Asp with depth is suggestive that l-Asp from cellular proteins underwent racemization during the burial, the d/l Asp ratio predicted by the sediment age (26 kyr for 3.4 m and 100 kyr for 5.8 and 14.8 m for conservative estimation), and assuming a constant average temperature of − 7.7 °C, varied from 0.36 to 0.89 (Fig. 1b). These predicted values were much greater than the observed values. Furthermore, the d/l Asp values of cellular proteins from the intact cell extract was 0.06 to 0.15, lower than that determined from the bulk sediment (Fig. 1). The d/l Asp of the bulk sediment must represent a balance between the much higher ratio of cells that died when the permafrost formed and the much lower ratio of microorganisms that remain viable and metabolically active whose existence was confirmed by Live/Dead cell staining (Additional file 1: Fig. S7).

### Reconstruction of MAGs and phylogeny

**Fig. 2:**
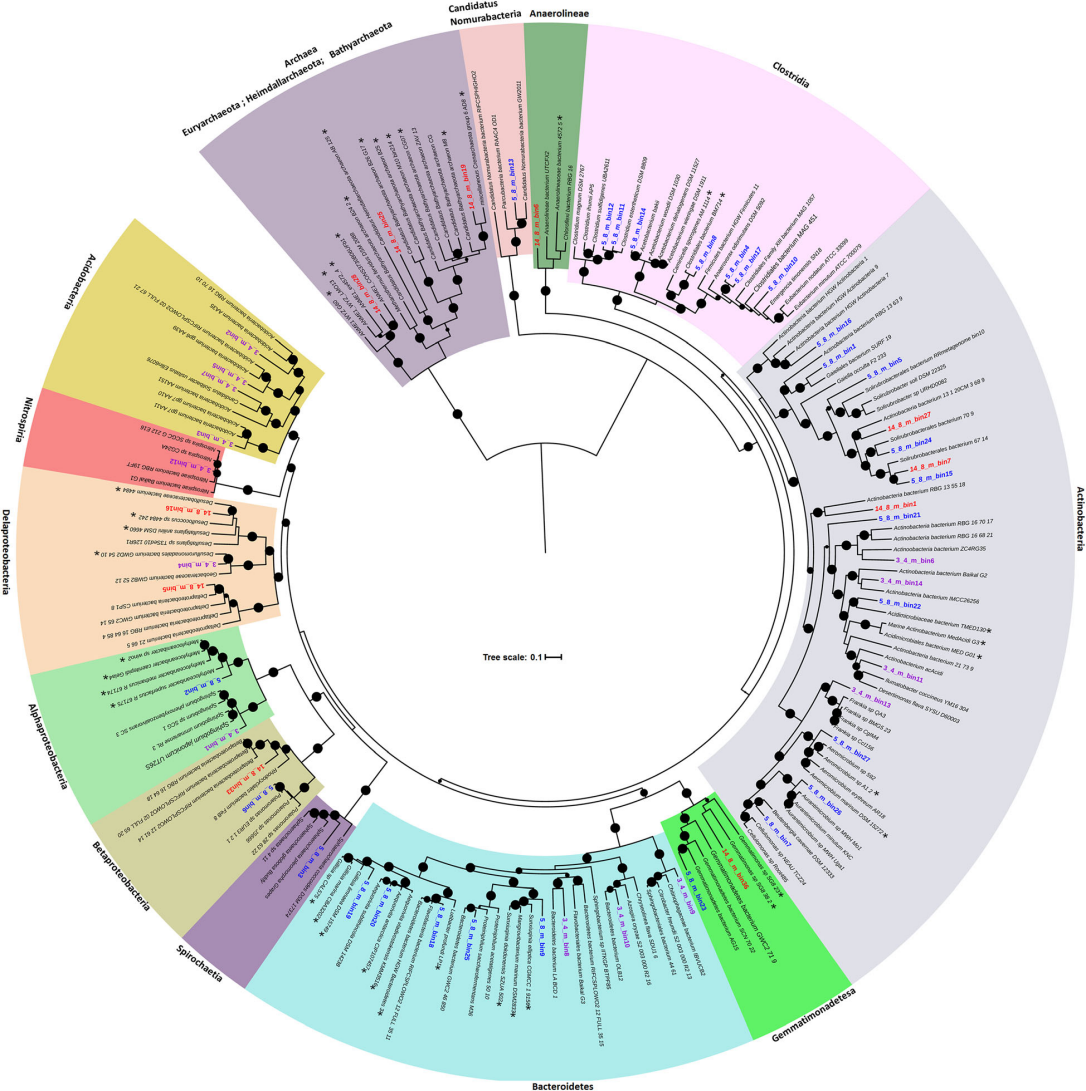
Phylogenetic tree of MAGs from 3.4 (purple), 5.8 (blue), and 14.8 (red) (m) and their closely related reference genomes from diverse environments. The maximum-likelihood phylogenomic tree was constructed based on up to 16 concatenated ribosomal proteins. The asterisks indicate those organisms originating from marine environments. The black dots represent bootstrap values > 70% (bootstrap values were generated from 1000 iterations)

By integrating three different algorithms and a consolidation strategy [40], a total of 144 medium- to high-quality MAGs [64] (> 50% complete and < 10% contamination) were recovered from the metagenomes obtained from 3.4, 5.8, and 14 m samples. Further re-assembly and refinement resulted in 52 MAGs (> 80% complete and < 10% contamination) that were selected for downstream analyses (Additional file 2: Table S1). The taxonomic distribution from phylogenomics (Fig. 2) and GTDB-Tk analysis (Additional file 3: Table S2) revealed that the reconstructed MAGs comprised 11 bacterial phyla (Candidatus *Nomurabacteria*, *Firmicutes*, *Actinobacteria*, *Proteobacteria*, *Nitrospirae*, *Acidobacteria*, *Gemmatimonadetes* and *Chloroflexi*, *Bacteroidetes*, and *Spirochaetes*) and 3 archaeal phyla (*Euryarchaeota*, *Bathyarchaeota*, and *Heimdallarchaeota*). MAGs from the Ice Complex of the Yedoma suite (3.4 m, Additional file 1: Fig. S2) were affiliated with the phyla *Actinobacteria* (4 genomes), *Acidobacteria* (4), *Bacteroidetes* (3), *Nitrospirae* (1), *Betaproteobacteria* (1), and *Deltaproteobacteria* (1). According to the relative abundance calculated from read-mapping (Additional file 1: Fig. S8), the Actinobacteria MAGs (3_4_m_bin11, 3_4_m_bin13, 3_4_m_bin14) were predominant whereas the *Bacteroidetes* (3_4_m_bin8 and 3_4_m_bin10) and *Acidobacteria* (3_4_m_bin7) were much less abundant in the Yedoma suite (Additional file 1: Fig. S8). The MAGs recovered from the 5.8 m sample from the marine Kon’kovaya suite were mainly from the phyla *Firmicutes* (7), *Actinobacteria* (10), *Bacteroidetes* (5), *Gemmatimonadetes* (1), and *Spirochaetes* (1) (Fig. 2 and Additional file 3: Table S2). The *Spirochaetes* (5_8_m_bin3), Actinobacteria (5_8_m_bin27 and 5_8_m_bin15), and several *Firmicutes* MAGs (5_8_m_bin4, 5_8_m_bin10, 5_8_m_bin12 and 5_8_m_bin17) were the most dominant bacteria (Additional file 1: Fig. S9). MAGs recovered from middle of the marine Kon’kovskaya suite at 14.8 m were closely related to microorganisms found in both marine and freshwater environments (Fig. 2). The archaeal MAGs belonging to *ANME-1*, *Bathyarchaeota*, and *Heimdallarchaeota* and bacterial MAGs affiliated to *Deltaproteobacteria*, *Chloroflexi*, and *Gemmatimonas* were exclusively present in the 14.8 m sample (Fig. 2). These marine lineages related to *Heimdallarchaeota* (14_8_m_bin25), *Bathyarchaeota* (14_8_m_bin19), *Chloroflexi* (14_8_m_bin6), and *Deltaproteobacteria* (14_8_m_bin16) were most abundant in the marine horizon particularly in the iDNA fraction (Additional file 1: Fig. S10).

### DNA repair and improved recovery of MAGs

DNA repair dramatically increased the percentage of unique reads (after removing duplicate reads) in the metagenomes from the older strata at 5.8 and 14.8 m for both iDNA and eDNA fractions (Additional file 1: Fig. S11). The increasing diversity of metagenomic reads suggested that the PreCR DNA repair enzymes enabled successful sequencing of damaged DNA fragments that could not be sequenced without PreCR DNA repair treatment. The similarity between 14.8 m iDNA and eDNA metagenomes and those from the 3.4 m freshwater permafrost dramatically increased after DNA repair (Additional file 1: Fig. S12). Furthermore, PCA analysis of the 16S rRNA genes derived from the iDNA and eDNA fractions with and without PreCR DNA repair revealed an increase in the similarity of the microbial communities of the 5.8 and 14.8 m marine samples with the 3.4 m freshwater permafrost sample (Additional file 1: Fig. S13). Such observations suggest that some species in the marine permafrost were similar to those in the freshwater permafrost but their DNA was damaged. The multidimensional scaling (MDS) analysis of the 16S rRNA genes also indicated that the microbial communities of 5.8 and 14.8 m samples were more diverse than that of the 3.4 m sample (Additional file 1: Fig. S13). The shift of the microbial community structure from the eDNA fraction after PreCR DNA repair toward that of the iDNA fractions, particularly in 14.8 m sample (Additional file 1: Fig. S13), suggests that extracellular DNA from past microbial communities were sequenced after PreCR DNA repair.

**Fig. 3:**
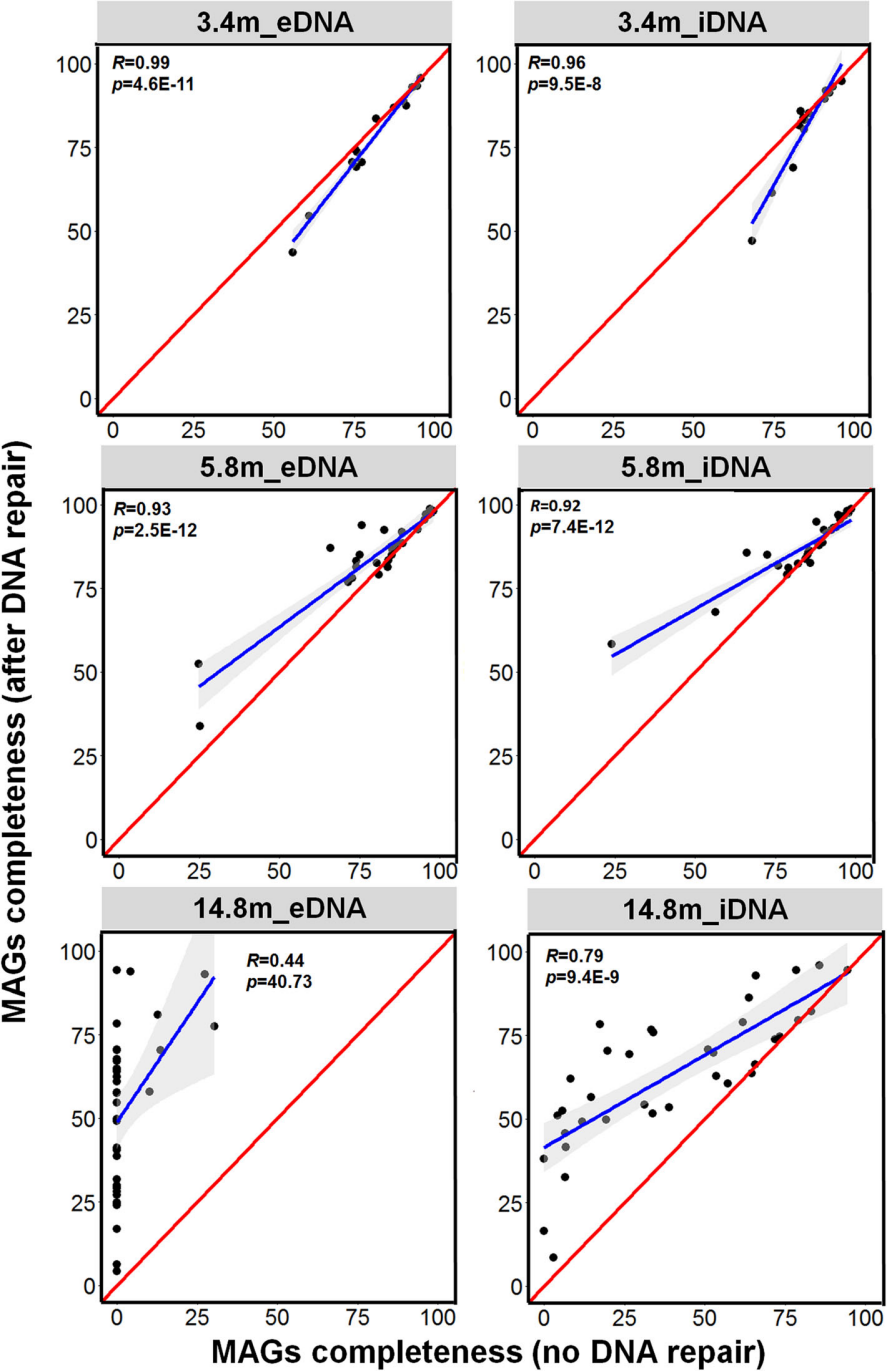
Comparison of genome completeness of MAGs recovered from individual metagenomes derived from iDNA and eDNA fractions with and without PreCR DNA repair. The blue line refers to the regression line from the correlation between MAGs completeness with and without PreCR DNA repair. The Pearson coefficient (*R*) and *p* value (*p*) are shown in each plot. The red line indicates the theoretical line by assuming no DNA damage and thus no positive effect on the MAGs completeness from PreCR DNA repair

**Fig. 4:**
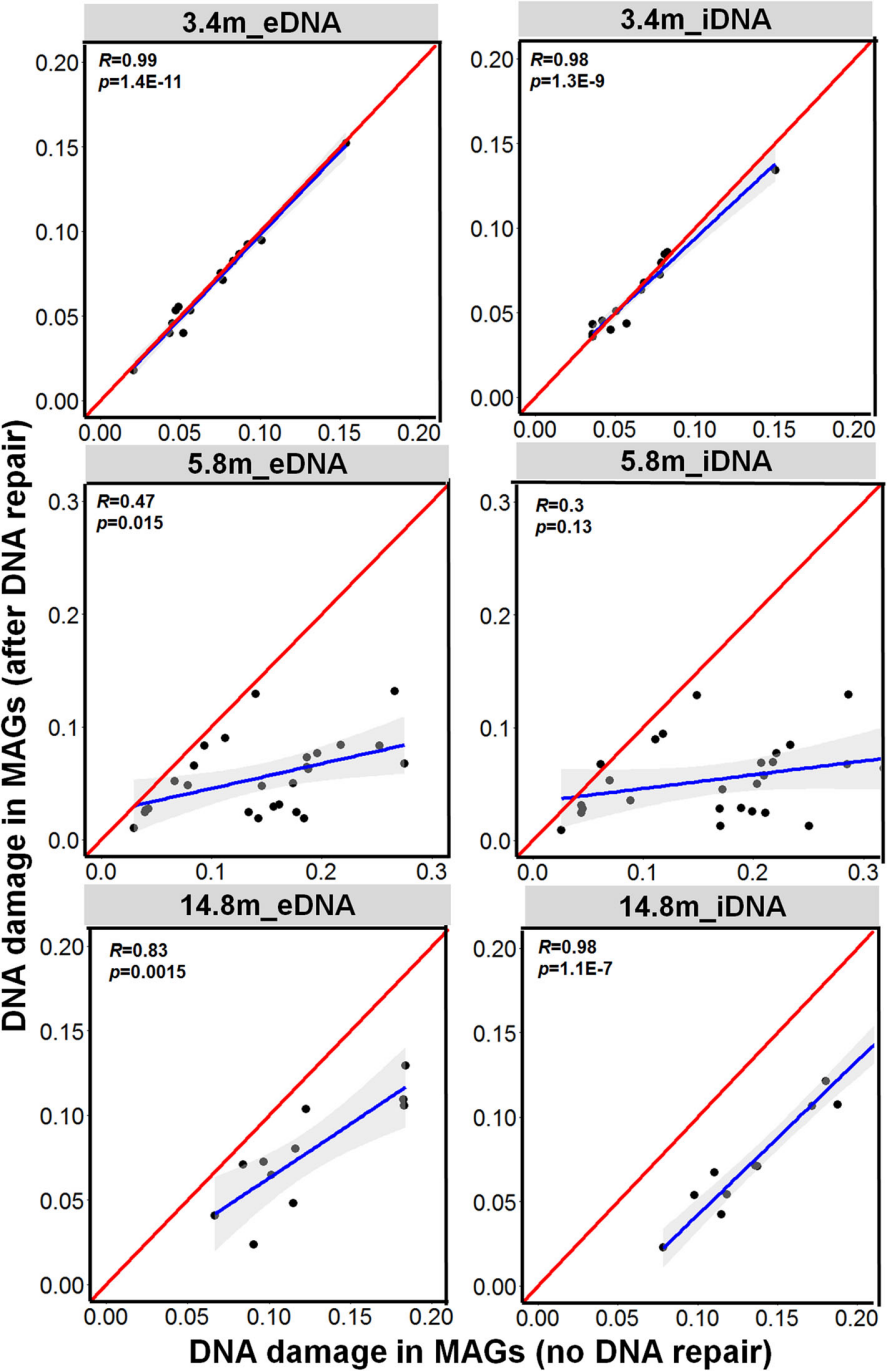
Comparison of DNA damage (cytosine deamination reflected by frequency of C to T substitution) of MAGs recovered from individual metagenomes derived from iDNA and eDNA fraction with and without PreCR DNA repair. The blue line refers to the regression line from the correlation between MAGs completeness with and without DNA repair. The Pearson coefficient (*R*) and *p* value (*p*) are shown in each plot. The red line indicates the theoretical line by assuming no DNA damage and no improvement of DNA damage from PreCR DNA repair

The effect of PreCR DNA repair on the MAGs derived from each DNA fraction was assessed by comparing the genome completeness (Fig. 3) and the severity of cytosine deamination for DNA treated with PreCR DNA repair versus those for DNA untreated by PreCR DNA repair (Fig. 4). If no effect of PreCR DNA repair on the derivation of MAGs, then the genome completeness and severity of cytosine deamination derived from all MAGs with and without DNA repair would overlap with the theoretical 1:1 line (red line in Figs. 3 and 4). Given that the metagenomic libraries with PreCR DNA repair yielded fewer reads than those without in vitro repair (Additional file 1: Fig. S14), the MAGs derived from under sequenced species (low completeness) would shift downward relative to the theoretical line (Fig. 3). The completeness of all MAGs from the 3.4 m sample overlapped with the theoretical line or slightly deviated toward the downside for both iDNA and eDNA fractions (Fig. 3). Furthermore, the frequency of cytosine deamination was relatively low (< 0.1) in the 3.4 m sample MAGs and followed the theoretical line with high correlation coefficient (0.98–0.99; Fig. 4). Therefore, the negligible effect of PreCR DNA repair on the 3.4 m sample is related to the minimal DNA damage and that all of the MAGs in the youngest layer might be recovered from lineages represented by presently living microorganisms that have been buried and frozen since 26 to 43 kyr ago. Among the 27 MAGs from the 5.8 m sample (Additional file 2: Table S1), PreCR DNA repair increased the genome completeness of several putatively aerobic *Actinobacteria* MAGs (Additional file 4: Table S3) and decreased the degree of DNA damage (Fig. 4 and Table S4) in most MAGs from both iDNA and eDNA fractions. However, the facultative or obligate anaerobic microorganisms affiliated with *Clostridiales*, *Spirochaeta*, *Bacteroidetes* showed much less DNA damage and thereby minimal impact of DNA repair on both genome completeness and frequency of cytosine deamination (Fig. 4 and Additional file 5: Table S4). Due to the highly damaged DNA recovered from the 14.8 m permafrost sediment, the PreCR DNA repair enzymes dramatically increased completeness of most MAGs and decreased DNA damage for all MAGs present in both iDNA and eDNA pools (Figs. 3 and 4, Additional files 3 and 4: Table S3 and S4). The positive effect of PreCR DNA repair was also revealed by the visualization of GC content and contig abundance in each MAG recovered from individual metagenomes of the iDNA and eDNA with and without PreCR DNA repair (Additional file 1: Fig. S15). However, several MAGs affiliated with *Chloroflexi* (14_8_m_bin6), *Bathyarchaeota* (14_8_m_bin19), and *Heimdallarchaeota* (14_8_m_bin25) showed minimal increase in genome completeness (falling on the theoretical line) in the iDNA fraction after PreCR DNA repair (Fig. 3 and Additional file 4: Table S3).

### Metabolic functions of recovered MAGs

**Fig. 5:**
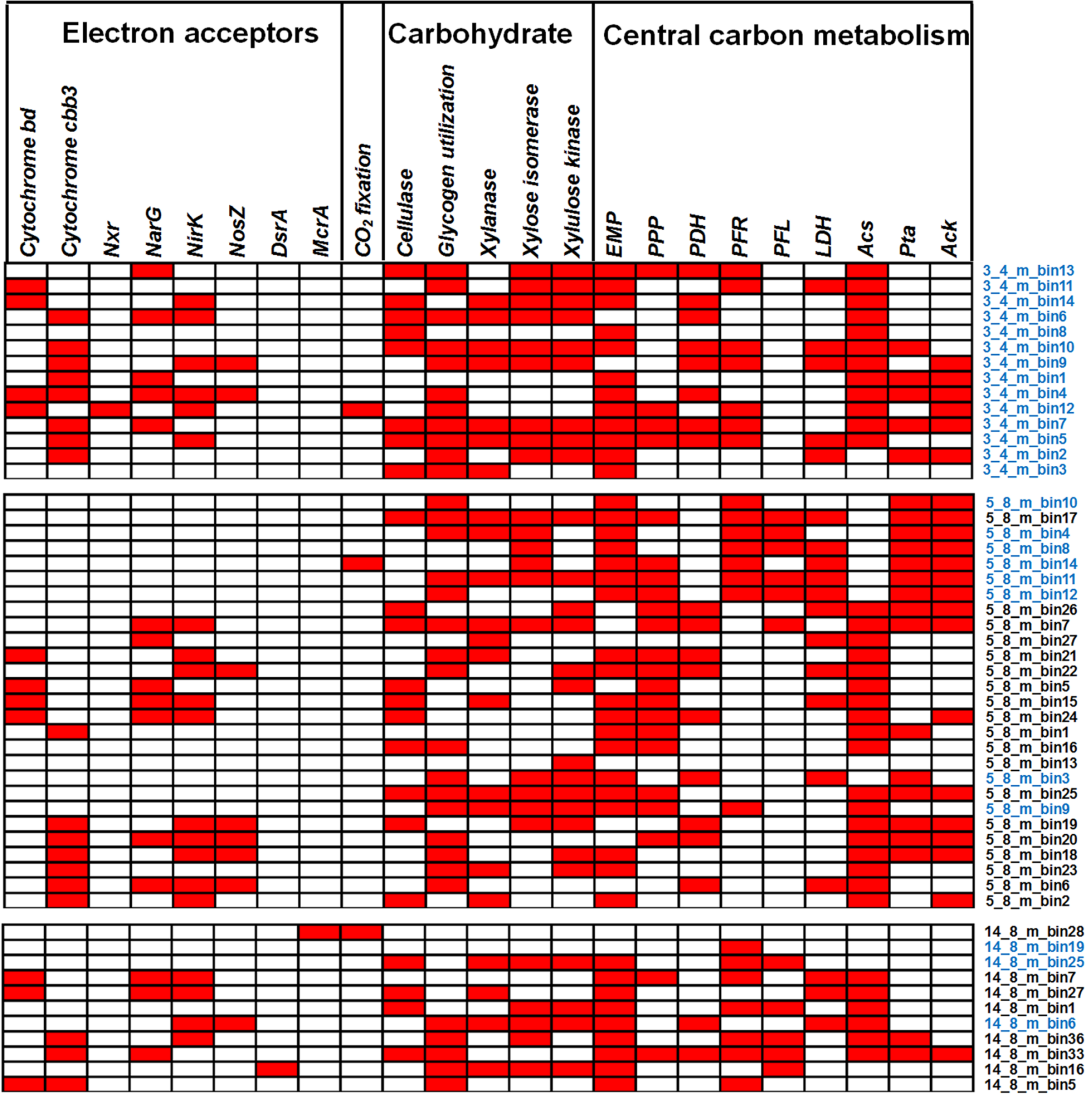
Key functional genes involved in carbon and energy metabolism that are present (red) in MAGs. Abbreviations: cytochrome bd terminal oxidase (Cytochrome bd), Cbb3-type cytochrome c oxidase (Cytochrome cbb3), nitrite oxidoreductase (*Nxr*), membrane bound nitrate reductase (*NarG*), nitrite reductase (*Nik*), nitrous oxide reductase (*NosZ*), dissimilatory sulfite reductase subunit A (*DsrAB*), Methyl-coenzyme M reductase (*McrA*), Embden-Meyerhof pathway (EMP), pentose phosphate pathway (PPP), pyruvate dehydrogenase (*PDH*), pyruvate:ferredoxin oxidoreductase (*PFR*), pyruvate formate lyase (*PFL*), Lactate dehydrogenase (*LDH*), Acetyl-CoA synthetase (*Acs*), phosphate acetyltransferase (*Pta*), and acetate kinase (*Ack*). Note: The CO_2_ fixation refer to the presence of genes involved either in reductive TCA cycle or Wood–Ljungdahl pathway and glycogen utilization pathway indicates that glycogen phosphorylase and glycogen debranching enzyme were identified in the genome. The MAGs highlighted in blue represent the potentially living microbial populations inferred from DNA damage in both iDNA and eDNA fractions

Three MAGs (3_4_m_bin12, 5_8_m_bin14, and 14_8_m_bin28) were predicted to be capable of autotrophic CO_2_ fixation via different pathways (Fig. 5). The *Nitrospira*-related MAG (3_4_m_bin12) is a nitrite-oxidizer that encodes key genes (ATP-citrate lyase, 2-oxoglutarate:ferredoxin oxidoreductase and fumarate hydratase) for the reductive citric acid cycle. The Wood–Ljungdahl pathway for CO_2_ fixation was identified in two MAGS (5_8_m_bin14 and 14_8_m_bin28) predicted to be involved in acetogenesis and anaerobic methane oxidation, respectively (Figs. 2 and 5). Metabolic reconstruction indicated that the majority of the MAGs (49 out of 52) recovered from three depths are capable of heterotrophic metabolism using a variety of terminal electron acceptors (Fig. 5). High affinity cytochrome terminal oxidases (cbb3- and bd-type) were identified in nearly all MAGs from the 3.4 m sample and many of the MAGs recovered from the 5.8 and 14.8 m samples (Fig. 5). Despite the potential of aerobic metabolism under microaerobic conditions in permafrost [8, 65], many of these MAGs also harbor genes involved in dissimilatory nitrate reduction and fermentative metabolism (Fig. 5). The MAGs from the 5.8 m sample were predominately fermentative organisms affiliated with Firmicutes and Spirochaetes. Several MAGs from the 14.8 m sample were predicted to be obligate anaerobes that can perform carbohydrate fermentation, sulfate reduction, and anaerobic methane oxidation. A large variety of carbohydrate-active enzymes within the groups of glycoside hydrolases (GHs), glycosyl transferases (GTs), carbohydrate esterases (CEs), and auxiliary activities (AAs) were identified from MAGs recovered from all three depths (Additional file 1: Fig. S16). Many of these enzymes from glycoside hydrolases and carbohydrate esterases play important roles in the degradation of various polysaccharides such as cellulose, chitin, glycogen, and peptidoglycan. For example, he enzymes (cellulase, xylanase, and glycogen phosphorylase) responsible for the breakdown of carbohydrate polymers were identified among MAGs affiliated with *Firmicutes*, *Acidobacteria*, *Actinobacteria*, and *Bacteroidetes* (Fig. 5). Monosaccharides and disaccharides can be further oxidized to pyruvate using Embden-Meyerhof pathway present in 40 MAGs and the pentose phosphate pathway present in 22 MAGs. Most of these fermentative anaerobes have the genetic potential to ferment pyruvate further into lactate, formate, and acetate (Fig. 5). Formate and acetate and propionate were detected throughout the core (Additional file 1: Fig. S4). The predominance of fermentative anaerobes affiliated with *Spirochaetes* (5_8_m_bin3) and *Firmicutes* (5_8_m_bin4, and 5_8_m_bin10, 5_8_m_bin12) coincided with the highest accumulation of low-molecular-weight organic acids at 5.8 m (Additional file 1: Fig. S4).

### Genetic potential for long-term survival strategies

**Fig. 6:**
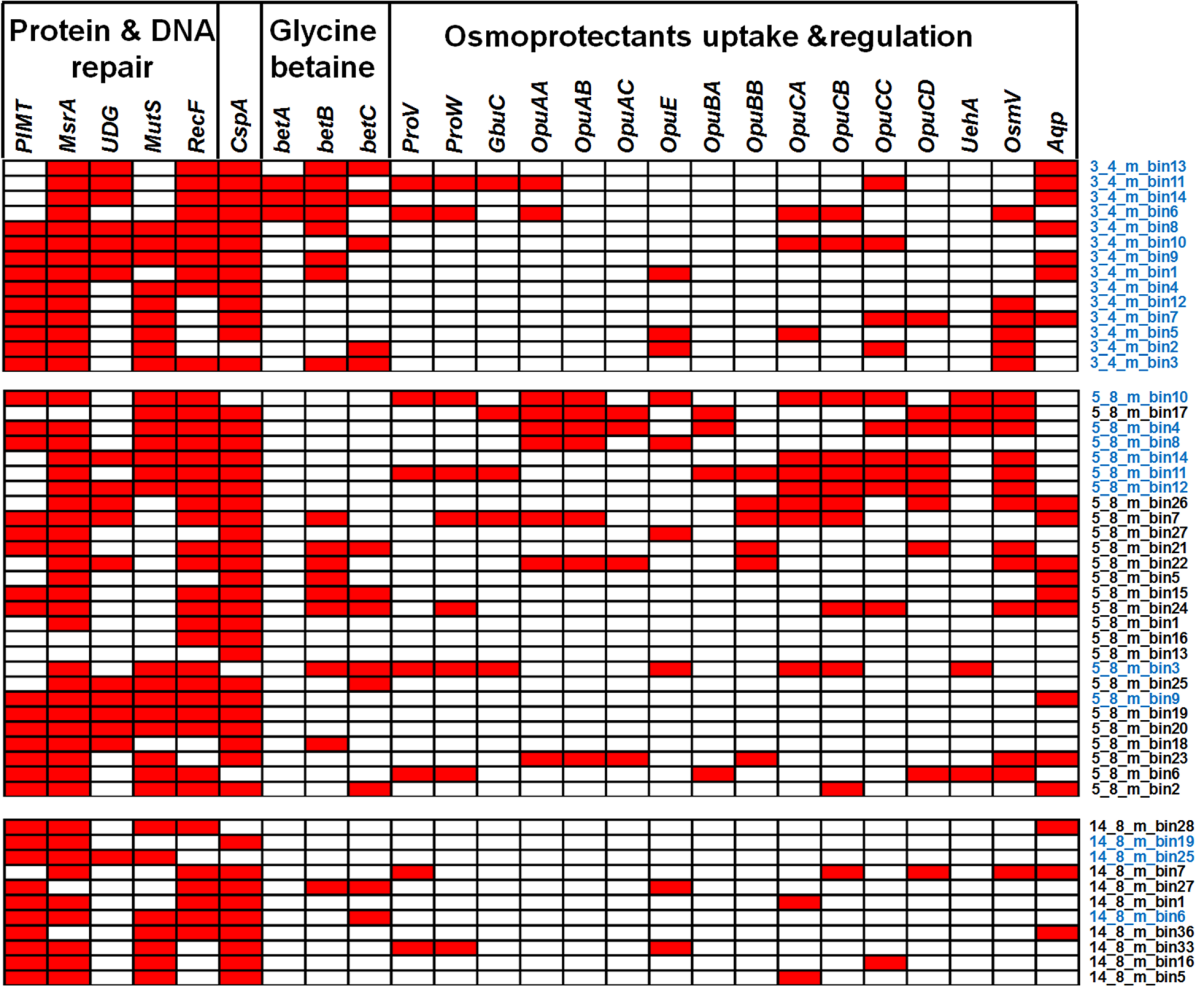
Key functional genes involved in the repair of DNA and protein damage and survival under cold, osmotic, and oxidative stresses present (red) in MAGs. Gene abbreviations: protein l-isoaspartyl/d-aspartyl o-methyltransferase (*PIMT*), methionine sulfoxide reductase (*MsrA*), uracil-DNA glycosylase (*UDG*), DNA mismatch repair protein (*MutS*), DNA replication and repair protein (*RecF*), cold shock protein (*CspA*), choline dehydrogenase (*betA*), betaine aldehyde dehydrogenase (*betB*), choline sulfatase (*betC*), glycine betaine/proline ABC transporter (*ProV*), glycine betaine/proline betaine transport system permease protein (*ProW*), Glycine betaine/carnitine transport binding protein (*GbuC*), osmoprotectant ABC transporter ATP-binding protein (*OpuAA*), glycine betaine transport system permease protein (*OpuAB*), glycine betaine-binding protein (*OpuAC*), osmoregulated proline transporter (*OpuE*), choline transport ATP-binding protein (*OpuBA*), choline transport system permease protein (*OpuBB*), carnitine transport ATP-binding protein (*OpuCA*), carnitine transport permease protein (*OpuCB*), carnitine transport binding protein (*OpuCC*), carnitine transport permease protein (*OpuCD*), ectoine/5-hydroxyectoine-binding periplasmic protein (*UehA*), osmoprotectant import ATP-binding proteins (*osmV*), and aquaporin (*Aqp*). The MAGs highlighted in blue represent the potentially living microbial populations inferred from DNA damage in both iDNA and eDNA fractions

The *PIMT* enzyme (Protein l-Isoaspartyl / d-Aspartyl O- Methyltransferase) has been implicated in protein repair by converting d-Asp back to l-Asp in all domains of life except for gram-positive bacteria [66]. The gene encoding *PIMT* was identified in 35 of the 52 MAGs including in MAGs belonging to gram-positive bacteria such as *Actinobacteria* and *Firmicutes* in all three depths (Fig. 6). Therefore, the presence of PIMT in most MAGs is consistent with its involvement in maintaining the low d/l Asp (Fig. 1) detected in the intact cell extracts [8]. The gene encoding methionine sulfoxide reductase (*MsrA*) that is important in coping with oxidative stress by reversing the oxidation of methionine in damaged proteins was found in 47 of the 52 bacterial and archaeal MAGs. Additionally, the uracil-DNA glycosylase, *MutS* and *RecF* genes responsible for DNA repair were identified in all but two of the MAGs (Fig. 6). Due to the anticipated DNA damage under frozen conditions, these universal DNA repair-related genes might play important roles in maintaining genomic integrity in the metabolically active cells through geological time. The genes for protection from cold shock, osmotic stress, and oxidative stress were identified in many of the MAGs (Fig. 6). The *Actinobacteria* MAGs recovered from three depths possess *betA*, *betB*, and *betC* for biosynthesis of the osmoprotectant glycine-betaine. Many of the *Firmicutes* MAGs from the 5.8 m sample lack the biosynthetic pathway for glycine-betaine, but harbor various genes for osmoregulation and uptake of osmoprotective compounds such as choline, glycine-betaine, proline, carnitine, and ectoine (Fig. 6). Given the perennial freezing temperature and high salinity in the marine horizon, the genetic machinery for uptake and synthesis of osmoprotectants enables the permafrost MAGs such as *Firmicutes*to survive under strong osmotic stress caused by the perennial freezing temperature and high salinity through geological time.

### Metaproteomics and in situ replication rates

**Fig. 7:**
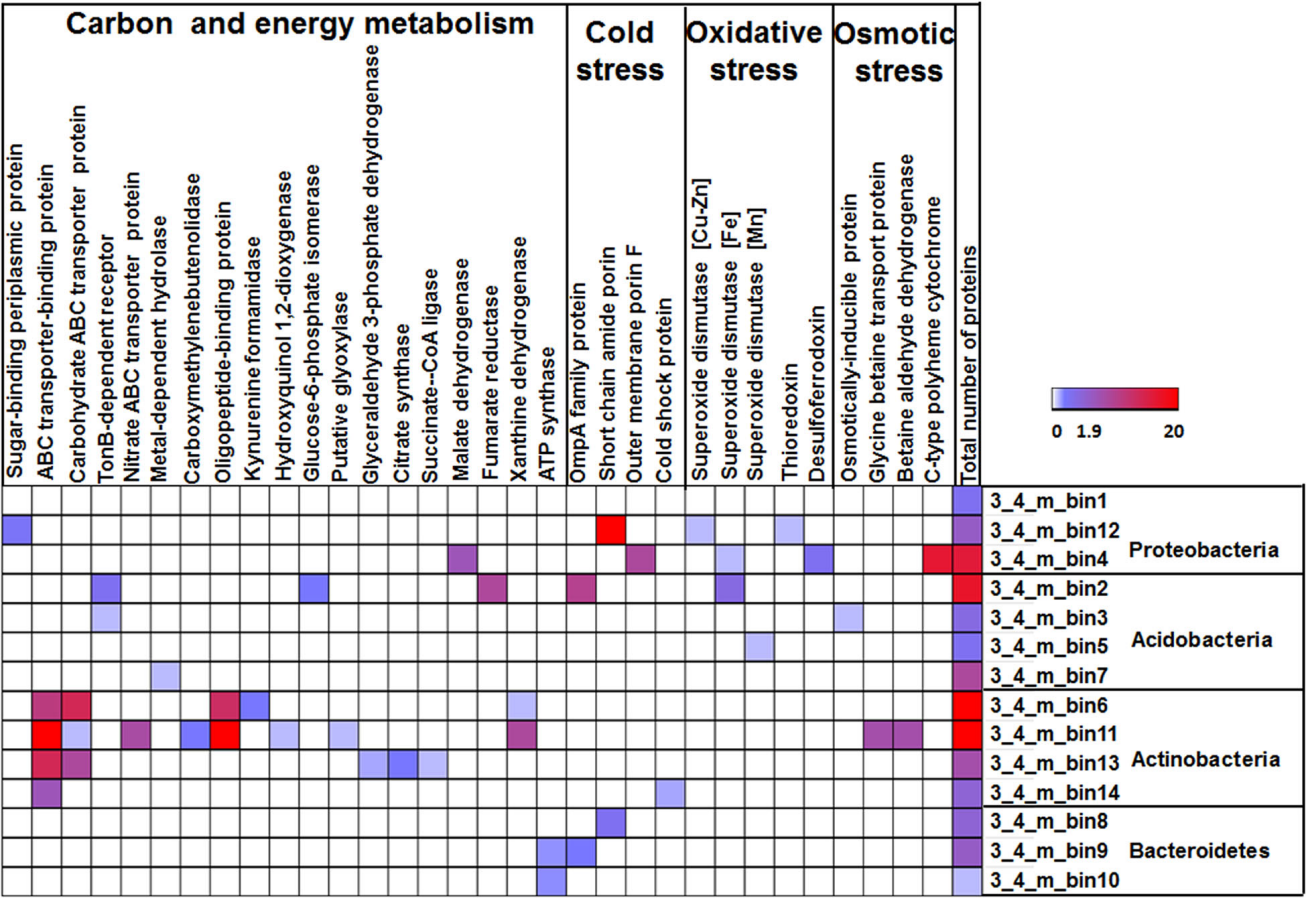
Relative abundance of proteins identified from each MAG recovered from 3.4 m sample. These proteins were involved in carbon and energy metabolism and coping with cold, osmotic, and oxidative stresses. The relative abundance refers to the balanced spectral counts of each identified protein, whereas total number of proteins indicated all proteins identified from each MAG

Despite the successful application of metaproteomics in active layers and modern permafrost at shallow depths [67, 68], no report has been published on metaproteomes from deeper and older permafrost due to the extremely low biomass and metabolic activity. The number of identified proteins dramatically decreased from the 3.4 m to the 14.8 m samples (Additional file 6: Table S5). Further genome-resolved metaproteomic analyses revealed that protein expression was identified from all 3.4 m sample MAGs (Fig. 7) and most MAGs of fermentative bacteria from the 5.8 m sample (Additional file 1: Fig. S17). Proteins related to carbohydrates utilization, TCA cycle, and ATP production were expressed from the MAGs recovered from the 3.4 and 5.8 m samples (Fig. 7 and Additional file 1: Fig. S17). Meanwhile, proteins potentially involved in coping with cold, oxidative, and osmotic stresses were also identified (Fig. 7 and Additional file 1: Fig. S17). The limited number of proteins identified from the 14.8 m sample (Additional file 1: Figs. S18-19) was mainly expressed in the marine-related phyla of *Heimdallarchaeota*, *Deltaproteobacteria*, and *Bathyarchaeota* (Fig. 2).

The limited number of identified proteins retrieved from the older permafrost samples (Table S5) reflects the lower overall biomass, but may also reflect lesser metabolic rates. We therefore used the metagenomic data and the Growth Rate Index (GRiD) [53] to estimate the in situ replication rates of the MAG members of the microbial populations. The GRiD values among the bacterial MAGs from three depths were low, varying from 1 to 1.52 (Additional file 1: Fig. S20). The low GRiD values suggest many bacterial cells have been dividing in a slow-growing mode. However, we caution that this method only provides meaningful insight into the actively growing microorganisms from ancient permafrost with minimal DNA damage (Figs. 3 and 4).

## Discussion

The high integrity of iDNA from the 3.4 m sample (Fig. S6) suggests that many microbial cells (Additional file 1: Fig. S5 and S7) have remained metabolically active and maintained genomic integrity at subzero temperatures [26, 27] for 26–43 kyr. The viability and metabolic activity of the microbial community was further supported by the lowest d/l Asp from cellular proteins (Fig. 1b) and presence of potential live cells from the sediment (Additional file 1: Fig. S7). The recovered MAGs from this layer were mainly from the phyla *Actinobacteria*, *Acidobacteria*, and *Bacteroidetes* (Fig. 2 and Additional file 3: Table S2). Of the 14 MAGs, 7 MAGs comprise *Nitrospirae* (3_4_m_bin12) and *Acidobacteria* (3_4_m_bin2 and 3_4_m_bin15) that were closely related to the permafrost-derived genomes from Stordalen Mire in northern Sweden [68] and Svalbard, Norway [65] (Additional file 4: Table S3). Remarkably, all 14 of the 3.4 m MAGs could be recovered from both iDNA and eDNA metagenomes with similar genome completeness and with no significant improvement in genome completeness was observed for the 3.4 m metagenomes after PreCR DNA repair treatment (Fig. 3 and Additional file 1: Fig. S8). Such observations suggest that the eDNA was released from recently expired cells of currently metabolizing microorganisms, as it has been reported in marine sediments [22, 23] and permafrost of Yedoma suite (~ 40 kyr old) from a different site in Siberia [8]. Due to the minimal DNA damage, PreCR DNA repair showed no impact on frequency of cytosine deamination in all 14 MAGs in both iDNA and eDNA fractions (Figs. 3 and 4). Furthermore, many proteins expressed from all genomes were related to carbon metabolism and potential survival mechanisms such as adaptation to cold, oxidative, and osmotic stresses (Fig. 7). Therefore, the genomes recovered from the Late Pleistocene Yedoma suite permafrost likely originated from metabolically active microbial populations based on the consistent evidence of minimal DNA damage, negligible impact of PreCR DNA repair on MAGs completeness, and proteins expressed from most MAGs.

The 5.8 m permafrost sample represents the layer close to the interface between the upper non-saline terrigenous layer and underlying marine horizon (Additional file 1: Fig. S2). The interface records the boundary of the Polar Ocean before the regression of the sea level between 100 and 120 kyr ago that led to freezing of the Kon’kovaya marine strata [2]. Although close relatives of 5 of the 27 MAGs from the 5.8 m sample have been detected in metagenomes or isolated from permafrost (Additional file 4: Table S3) or freshwater environments [2, 17, 31, 68, 69], 8 MAGs from *Clostridia* (1), *Actinobacteria* (2), *Alphaproteobacteria* (1), and *Bacteroidetes* (4) were closely related to microbial species found in coastal sediments and seawater (Fig. 2). Since pores within these frozen aquatic sediments are completely filled with ice, migration of microorganisms within the stratum and microbial penetration from the top seasonally thawed active layer are prevented through geological time [2]. Therefore, the coexistence of freshwater and marine microorganisms in the 5.8 m sample reflects the stratification of this layer where brackish water might have mixed with precipitation before the permafrost formation (Fig. S2). The MAGs related to *Spirochaetes* (5_8_m_bin3) and *Clostridia* (bins 4, 8, 10–12, 14, and 17) were overwhelmingly predominant in the 5.8 m sample (Additional file 1: Fig. S9). A *Spirochaetes* species closely related to that of the 3.4 m sample has been isolated from methanogen enrichments derived from Holocene permafrost of floodplain bogs in Siberia [70]. Several species of *Clostridia* have been isolated from various permafrost sediments and cryopegs embedded in the Kon’kovaya suite marine strata [2, 17, 31, 69]. Given the typical low redox potential (down to − 256 mV SHE) in these deeper, older layers [71], the prevailing anaerobic, fermentative capability of the *Clostridia* MAGs of the 5.8 m sample is supported by the enrichment of genes for mixed acid fermentation producing acetate and formate from carbohydrates (Fig. 5). Current metabolic activity of the *Clostridia* MAGs is suggested by the expression of several genes related to carbohydrate metabolism in the metaproteome (Additional file 1: Fig. S16) and the high concentrations of acetate and formate in the 5.8 m sample pore water (Additional file 1: Fig. S4). Moreover, 3 of the 7 Clostridia MAGs encode a suite of genes (Fig. 6) that were actively expressed to cope with cold, oxidative, and osmotic stresses (Additional file 1: Fig. S17). The PreCR DNA repair had negligible impact on the completeness and frequency of cytosine deamination in the *Clostridia* MAGs from obligate anaerobic lineages (Additional files 4 and 5: Table S3 and S4). Therefore, these groups of anaerobic microbes have become adapted to the cryogenic and anoxic environments in this ancient frozen marine horizon. The other MAGs affiliated with the phyla *Actinobacteria*, *Proteobacteria*, and *Bacteroidetes* were mostly predicted to be obligate or facultative aerobes. The PreCR DNA repair dramatically increased the completeness of most of these MAGs for both iDNA and eDNA fractions (Figs. 3 and 4). Furthermore, very few proteins were identified in the metaproteomes of the 5.4 m sample (Additional file 1: Fig. S17) that were related to these MAGs despite the presence of cold-adaptation genes in some of these MAGs (Fig. 6). Therefore, these MAGs might represent past obligate or facultative aerobic microbial populations that have died upon deposition and freezing 100–120 kyr ago.

The dominant archaeal MAGs of the 14.8 m permafrost sample belonged to *ANME-1* (14_8_m_bin28), *Bathyarchaeota* (14_8_m_bin19), and *Heimdallarchaeota* (14_8_m_bin25) and the bacterial MAGs were affiliated with the *Deltaproteobacteria* (14_8_m_bin16), *Chloroflexi* (14_8_m_bin6) and *Gemmatimonadetes* (14_8_m_bin36). These MAGs were exclusively present in this 100–120 kyr marine strata (Fig. 2 and Additional file 1: Fig. S10) and showed high similarity to microbial lineages from deep-sea hydrothermal vents [72] and other marine sediments [73]. Furthermore, the genome completeness from these anaerobic marine lineages remained little changed in the iDNA fraction (Fig. 3 and Additional file 4: Table S3). The metaproteomics data showed that most of the identified proteins were associated with archaea and *Chloroflexi* (Fig. S18). The most proteins were identified from *Heimdallarchaeota* (14_8_m_bin25) when searched against all recovered MAGs. Therefore, the *Heimdallarchaeota*, *Deltaproteobacteria*, and *Chloroflexi* MAGs originating from Kon’kovaya suite marine sediments might have remained metabolically active in the cryogenic environment to maintain genomic integrity for 100–120 kyr. The remaining MAGs from 14.8 m were phylogenetically close to *Actinobacteria* and *Betaproteobacteria* groups detected in non-marine subsurface sediments and groundwater [74]. Many of these MAGs belong to the same genus or species that were also recovered from the 3.4 m and 5.8 m samples (Fig. 2 and Additional file 7: Table S6). Moreover, two other Actinobacteria MAGs (14_8_m_bin1 and 14_8_m_bin31) were closely related to genomes recovered from non-saline, modern permafrost from Stordalen Mire in northern Sweden [68] (Additional file 8: Table S7). Therefore, these MAGs represent microorganisms from non-saline permafrost environments that were likely transported through the water column and deposited into the coastal marine sediment over time before the Kon’kovaya suite froze. These microorganisms might not have survived the anoxic and highly saline pore water due to the requirement of O_2_ for respiration and the lack of most essential genes for coping with osmotic stress (Figs. 5 and 6). Indeed, the DNA from these MAGs was highly damaged in both iDNA and eDNA fractions and the MAGs were only recovered after PreCR DNA repair. Therefore, these MAGs likely are non-indigenous, dead microorganisms trapped in the marine strata and represent past Middle Pleistocene microbial populations that predate 100–120 kyr.

The demarcation between past and living microorganisms in ancient permafrost has important implications in global permafrost thaw and climate change. The widely distributed coastal permafrost in Siberian Arctic Shelf and other regions represent a large pool of ancient organic carbon [75, 76]. These ancient permafrost sediments along coastline of the East Siberian Arctic Shelf are susceptible to rapid erosion and degradation due to global warming [77]. Previous studies have focused on the response of microorganisms to thawing of modern near-surface permafrost [67, 68, 78, 79]. Due to thermal collapse and erosion of these carbon-rich Plio/Pleistocene coastline permafrost sediments, it is important to understand the metabolic status of the buried microorganisms in the deeper, older permafrost along the Arctic coastline across the Beringian region. The predominant heterotrophic aerobes (Fig. 5) from the upper layer (3.4 m) represent live microorganisms that can accelerate greenhouse gas emissions via decomposition of the trapped ancient organic matter in thawing permafrost. Since many obligate or facultative aerobes in the deeper, marine strata at 5.8 and 14.8 m represent the dead remains of Middle Pleistocene microbial populations, they will play no role in carbon cycling during thawing of these deeper, older permafrost deposits. However, the obligate and facultative anaerobes in these layers can contribute to CO_2_ and CH_4_ emissions via fermentation of carbohydrates during permafrost thaw. Since only a small fraction of microorganisms have survived in the sub-freezing conditions in the deeper, marine horizon (Additional file 1: Figs. S5 and S7) through geological time, the impact of permafrost thaw on greenhouse gas emission and climate change might be less in the deeper permafrost. Our work demonstrates that the discrimination of fossil versus living microorganisms is critical to understanding the role of buried microbes in carbon cycling upon permafrost thaw as a result of global warming.

## Conclusions

Our work couples PreCR DNA repair protocols with genome-resolved metagenomics to reconstruct MAGs from the fossil and living microorganisms entrapped in ancient frozen sediment that captures a 70 kyr time span and geochemical gradient. Different MAGs were recovered at each depth to record the diversity and metabolic potential of indigenous microbial populations in ancient permafrost along a geochemical gradient. The genomic insights into the past and present microbial populations expand our understanding about the microbial successions from the past to the present-day in ancient permafrost. The recovery of genomes from chronosequence-based permafrost also enhances our fundamental understanding about adaptive strategies and long-term survivability of present living microorganisms in young to ancient permanently frozen sediments. Moreover, the reconstruction of metabolic pathways from paleogenomes provides insight into the paleoenvironment and previous biogeochemical processes. Due to the exceptional preservation of eDNA under perennial frozen conditions, our results highlighted that eDNA should be considered when applying sequencing-based techniques to understanding microbial ecology particularly in ancient permafrost of much older geological ages. The combined strategies in this study can be a useful and effective tool for studying paleogenomics of microorganisms in other ancient environments such as deep-sea sediments and cave deposits where fossil DNA sequences might be well preserved [23, 24]. Furthermore, our findings can help constrain the search for past and extant extraterrestrial life over geological time scale in permafrost and ice deposits on Mars [80] and Europa [81].

## Supplementary Information

**Additional file 1: Figure S1.** Geographic location of the sampling site at Cape Chukochii near the East Siberian Sea coast. **Figure S2.** Image of the drilling site and the schematic of the sediment core (~22 m) from borehole Ch1-17. The red stars indicate the depth of the sediment samples (3.4, 5.8 and 14.8 m, meters below land surface) that were selected for metagenomic sequencing of iDNA and eDNA with and without DNA repair. **Figure S3** Temperature and geochemistry profiles of the permafrost sediment collected at various depths from borehole Ch1-17. **Figure S4.** Concentration of low-molecular-weight organic acids in the permafrost sediment collected at various depths from borehole Ch1-17. **Figure S5** Yield of iDNA and eDNA fractions from ancient permafrost sediment at 3.4, 5.8 and 14.8 m. The green bars represent the estimated cell numbers from the intracellular DNA fraction by assuming 2×10^-15^ g DNA/cell. **Figure S6.** Size distribution of DNA fragments in iDNA (3.4i, 5.8i and 14.8i) and eDNA (3.4e, 5.8e and 14.8e) fractions from ancient permafrost sediment at 3.4, 5.8 and 14.8 m. The top peaks at 10380 bp for 3.4iDNA and 3.4eDNA sampels were cropped out due to the much higher concentration**. Figure S7.** Live/Dead cell staining of separated cells from ancient permafrost sediments at 3.4 (top), 5.8 (middle) and 14.8 m (bottom). The green stained live cells by Syto9 are shown in the left panel whereas the red stained dead cells are depicted in the right panel. **Figure S8.** Relative abundance of MAGs from each metagenome of iDNA and eDNA at 3.4 m with and without PreCR DNA repair. The scale bar indicates the relative abundance of each MAG normalized to the individual sample size as genome copies per million reads**. Figure S9.** Relative abundance of MAGs from each metagenome of iDNA and eDNA at 5.8 m with and without PreCR DNA repair. The scale bar indicates the relative abundance of each MAG normalized to the individual sample size as genome copies per million reads. **Figure S10.** Relative abundance of MAGs from each metagenome of iDNA and eDNA at 14.8 m with and without PreCR DNA repair. The scale bar indicates the relative abundance of each MAG normalized to the individual sample size as genome copies per million reads. **Figure S11.** Percentage of unique reads in each metagenome generated from iDNA and eDNA fractions of the 3.4, 5.8 and 14.8 m samples with and without PreCR DNA repair. **Figure S12.** Heatmap of the global similarity of each iDNA and eDNA derived metagenomes from the 3.4, 5.8 and 14.8 m samples with and without PreCR DNA repair. The similarity matrix was calculated from the similarity of reads in each metagenome. The scale bar represents the normalized percentage of similarity between two metagenomes with respect to the total number of reads in each metagenome. **Figure S13.** Principal coordinate analyses (PCoA) of weighted UniFrac distances derived from the microbial community based on the 16S rRNA genes retrieved from each metagenomic dataset of iDNA and eDNA fraction with and without PreCR DNA repair. **Figure S14.** Number of quality-filtered reads in each metagenome generated from iDNA and eDNA fractions extracted from ancient permafrost sediment at 3.4, 5.8 and 14.8 m with and without PreCR DNA repair. **Figure S15.** Plot of GC content and contig abundance in each MAG recovered from individual metagenomes of the iDNA and eDNA extracted from the 14.8 m with and without PreCR DNA repair. **Figure S16.** Groups of carbohydrates active enzymes identified in MAGs recovered from three depths at 3.4, 5.8 and 14.8 m, respectively. The abbreviations for the enzymes classes are as follow: The glycoside hydrolases (GHs), glycosyl transferases (GTs), carbohydrate esterases (CEs) and auxiliary activities (AAs). The relative abundance represents the number of carbohydrates active enzymes identified in each specific subgroup. **Figure S17.** Relative abundance of proteins identified in the metaproteome from each MAG recovered from the 5.8 m sample. These proteins were involved in carbon and energy metabolism and coping with cold, osmotic and oxidative stresses. The scale bar indicates the balanced spectral counts of proteins. **Figure S18.** Number of identified proteins in the metaproteomic dataset at 14.8 m when all genes from the metagenome were used as database for search. **Figure S19.** Number of identified proteins from each MAG recovered from 14.8m. **Figure S20** GRiD measurement of bacterial MAGs from the iDNA metagenomic datasets from ancient permafrost sediment at 3.4, 5.8 and 14.8m. The criterion of valid GRiD values (*dnaA/ori* and *ter/dif* ratios > 0.8) for each MAGs was selected according to the output results from the GRiD tool.

**Additional file 2: Table S1.** Statistical summary for MAGs recovered from permafrost sediment samples at 3.4, 5.8 and 14.8 m. Note: CDS refers to protein coding sequence.

**Additional file 3: Table S2.** Taxonomic classification of middle to high-quality permafrost MAGs based on the GTDB-Tk tool.

**Additional file 4: Table S3.** Complteness of MAGs recovered iDNA and eDNA fractions from 14.8m with and without DNA repair.

**Additional file 5: Table S4.** Frequency of cytosine deamination from MAGs recovered iDNA and eDNA fractions from 3.4m with and without DNA repair.

**Additional file 6: Table S5.** Summary of identified proteins from ancient permafrost at 3.4, 5.8 and 14.8m.

**Additional file 7: Table S6.** Closely related MAGs shared at three different depths.

**Additional file 8: Table S7.** MAGs that are closely related to genomes recovered from permafrost-associated soils. Note: MAGs with over 50% completeness were considered.

**Additional file 9.** The scripts used for the analyses in this study.

## Data Availability

All raw sequences from the metagenomes were deposited in NCBI SRA under the BioProject PRJNA680161. All genome sequences have been made publicly available on GenBank under the accession numbers (SAMN16871416–SAMN16871467). The mass spectrometric RAW files were deposited to the ProteomeXchange Consortium via the PRIDE partner repository (identifier number: PXD022683). The scripts used for the analyses in this study were provided in Additional file 9 in the supplementary materials.
