# Supplementary material for: Genomic reconstruction of fossil and living microorganisms in ancient Siberian permafrost
Source: Microbiome. 2021 May 17;9:110. doi: 10.1186/s40168-021-01057-2 (PMC8130349; doi:10.1186/s40168-021-01057-2)
Supplement: Supplementary file 2 — Additional file 1: Figure S1. Geographic location of the sampling site at Cape Chukochii near the East Siberian Sea coast. Figure S2. Image of the drilling site and the schematic of the sediment core (~22 m) from borehole Ch1-17. The red stars indicate the depth of the sediment samples (3.4, 5.8 and 14.8 m, meters below land surface) that were selected for metagenomic sequencing of iDNA and eDNA with and without DNA repair. Figure S3 Temperature and geochemistry profiles of the permafrost sediment collected at various depths from borehole Ch1-17. Figure S4. Concentration of low-molecular-weight organic acids in the permafrost sediment collected at various depths from borehole Ch1-17. Figure S5 Yield of iDNA and eDNA fractions from ancient permafrost sediment at 3.4, 5.8 and 14.8 m. The green bars represent the estimated cell numbers from the intracellular DNA fraction by assuming 2×10-15 g DNA/cell. Figure S6. Size distribution of DNA fragments in iDNA (3.4i, 5.8i and 14.8i) and eDNA (3.4e, 5.8e and 14.8e) fractions from ancient permafrost sediment at 3.4, 5.8 and 14.8 m. The top peaks at 10380 bp for 3.4iDNA and 3.4eDNA sampels were cropped out due to the much higher concentration. Figure S7. Live/Dead cell staining of separated cells from ancient permafrost sediments at 3.4 (top), 5.8 (middle) and 14.8 m (bottom). The green stained live cells by Syto9 are shown in the left panel whereas the red stained dead cells are depicted in the right panel. Figure S8. Relative abundance of MAGs from each metagenome of iDNA and eDNA at 3.4 m with and without PreCR DNA repair. The scale bar indicates the relative abundance of each MAG normalized to the individual sample size as genome copies per million reads. Figure S9. Relative abundance of MAGs from each metagenome of iDNA and eDNA at 5.8 m with and without PreCR DNA repair. The scale bar indicates the relative abundance of each MAG normalized to the individual sample size as genome copies per million reads. Figure S10. Rela [file 40168_2021_1057_MOESM1_ESM.docx]

**Supplementary information for**

**Genomic reconstruction of fossil and living microorganisms in ancient Siberian permafrost**

Renxing Liang^1^**^*^**, Zhou Li^2^, Maggie C.Y. Lau Vetter^1#^, Tatiana A. Vishnivetskaya^3,4^, Oksana G. Zanina^4^, Karen G. Lloyd^3^, Susan Pfiffner^3^, Elizaveta M. Rivkina^4^, Wei Wang^5^, Jessica Wiggins^5^, Jennifer Miller^5,^ Robert L Hettich^2^, Tullis C. Onstott^1^

^1^Princeton University, Princeton, NJ, USA

^2^Chemical Sciences Division, Oak Ridge National Laboratory, Oak Ridge, TN, USA

^3^University of Tennessee, Knoxville, TN, USA

^4^Institute of Physicochemical and Biological Problems in Soil Science, Russian Academy of Sciences, Pushchino, Moscow Region, Russia

^5^Genomics Core Facility, Princeton University, Princeton, NJ, USA


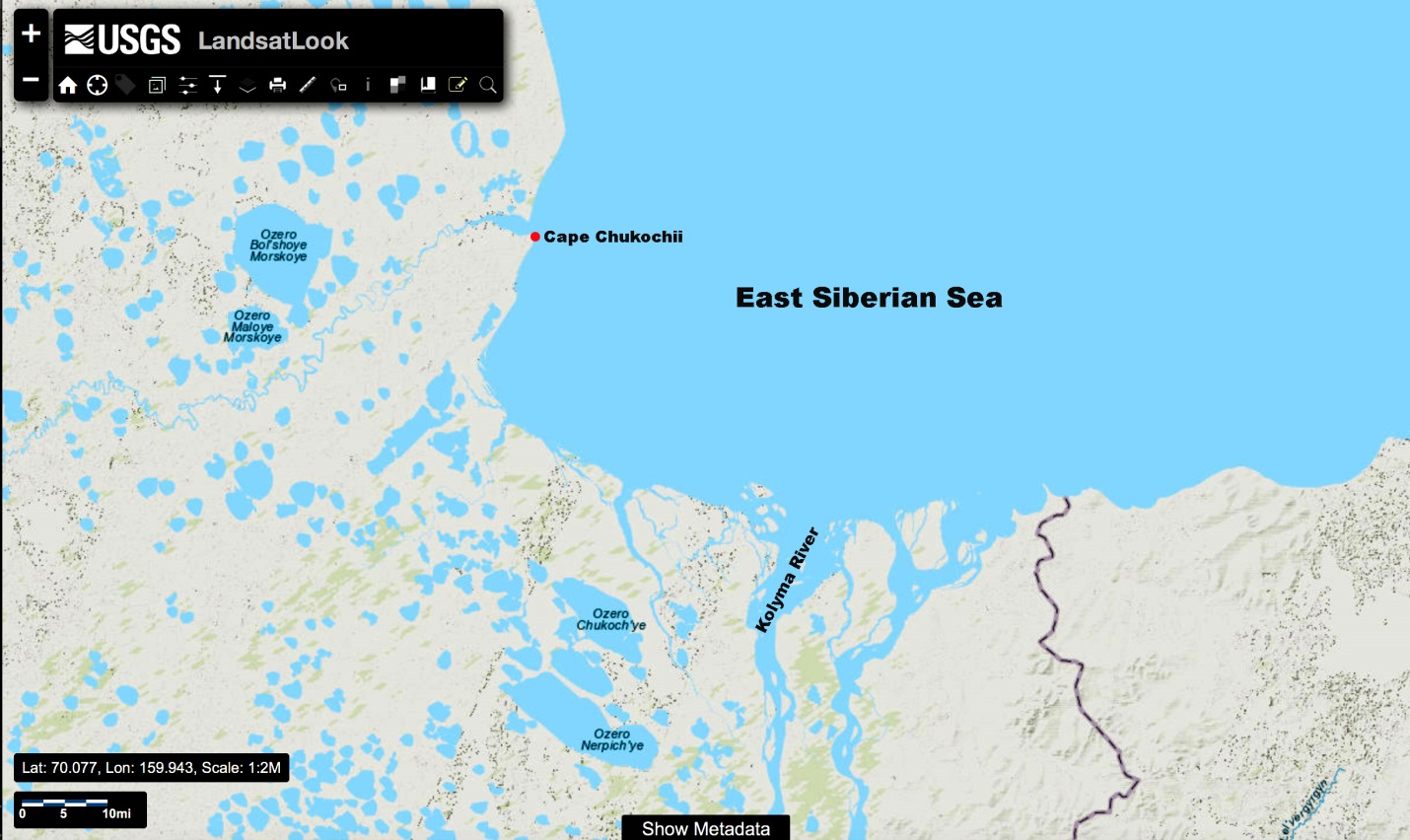


**Figure S1** Geographic location of the sampling site at Cape Chukochii near the East Siberian Sea coast.


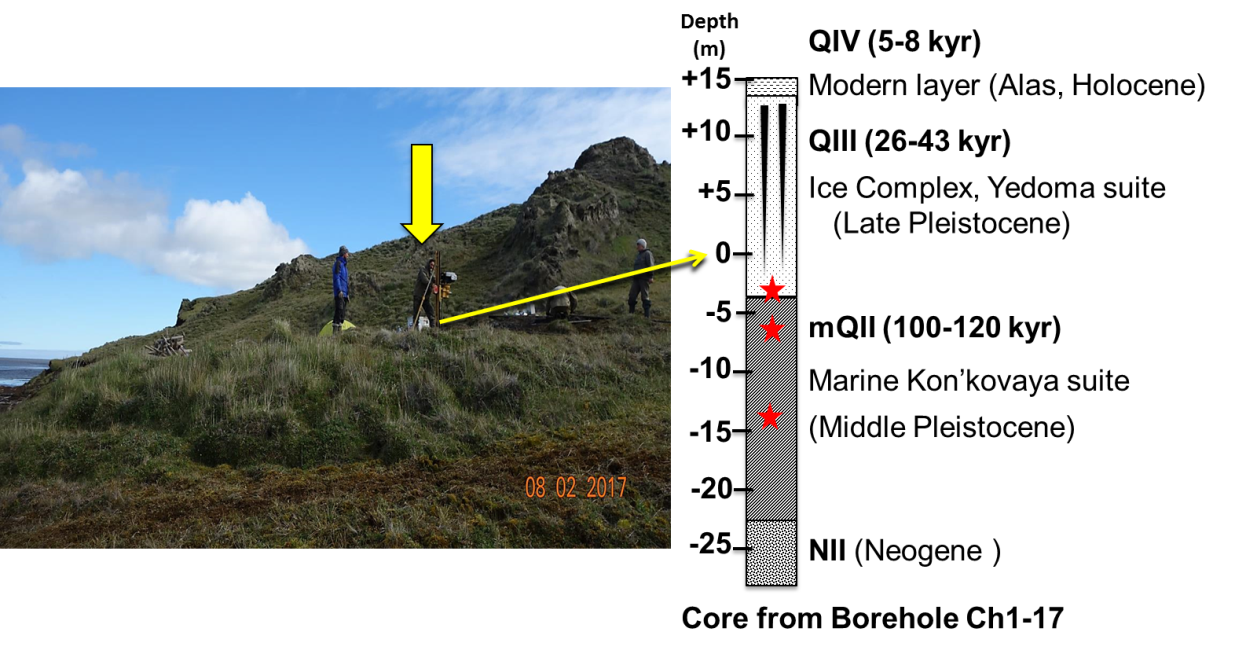


**Figure S2** Image of the drilling site and the schematic of the sediment core (~22 m) from borehole Ch1-17. The red stars indicate the depth of the sediment samples (3.4, 5.8 and 14.8 m, meters below land surface) that were selected for metagenomic sequencing of iDNA and eDNA with and without DNA repair.


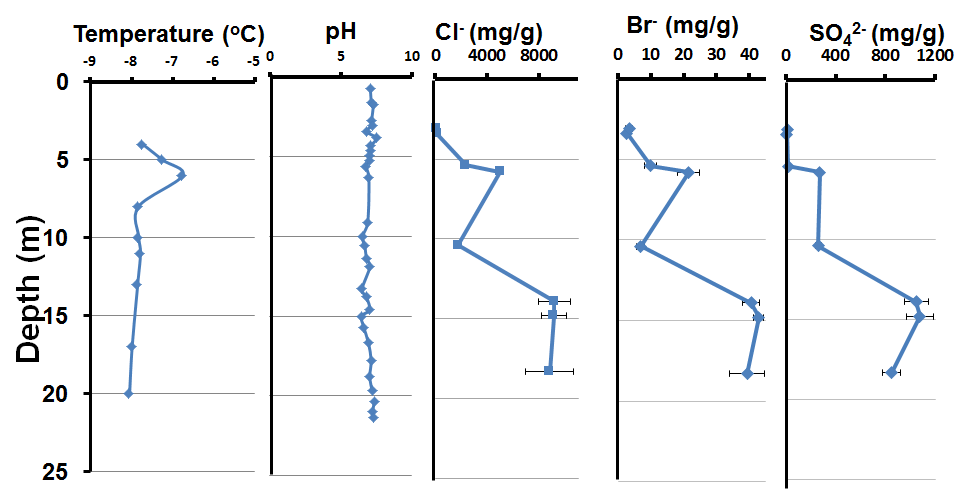


**Figure S3** Temperature and geochemistry profiles of the permafrost sediment collected at various depths from borehole Ch1-17.


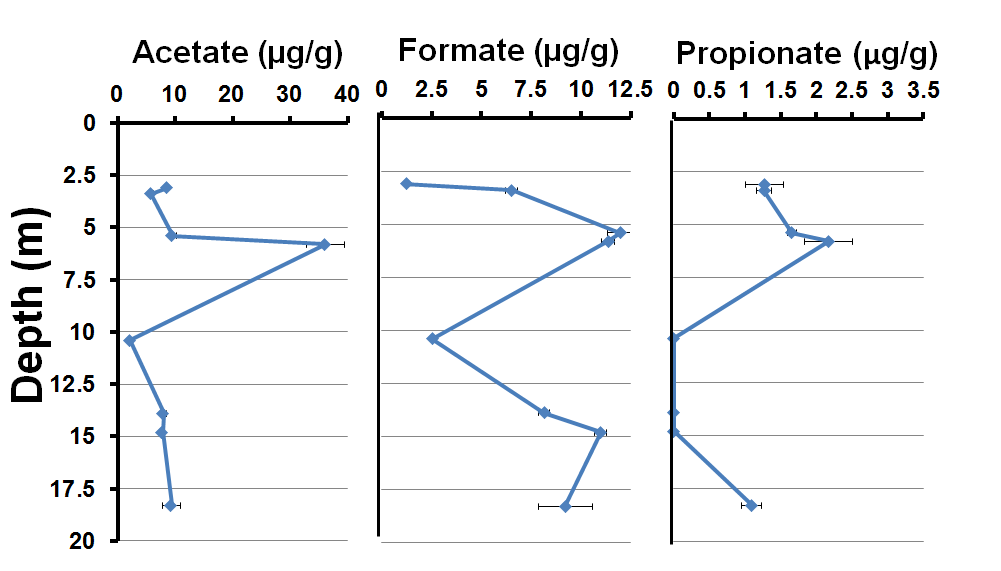


**Figure S4** Concentration of low-molecular-weight organic acids in the permafrost sediment collected at various depths from borehole Ch1-17.


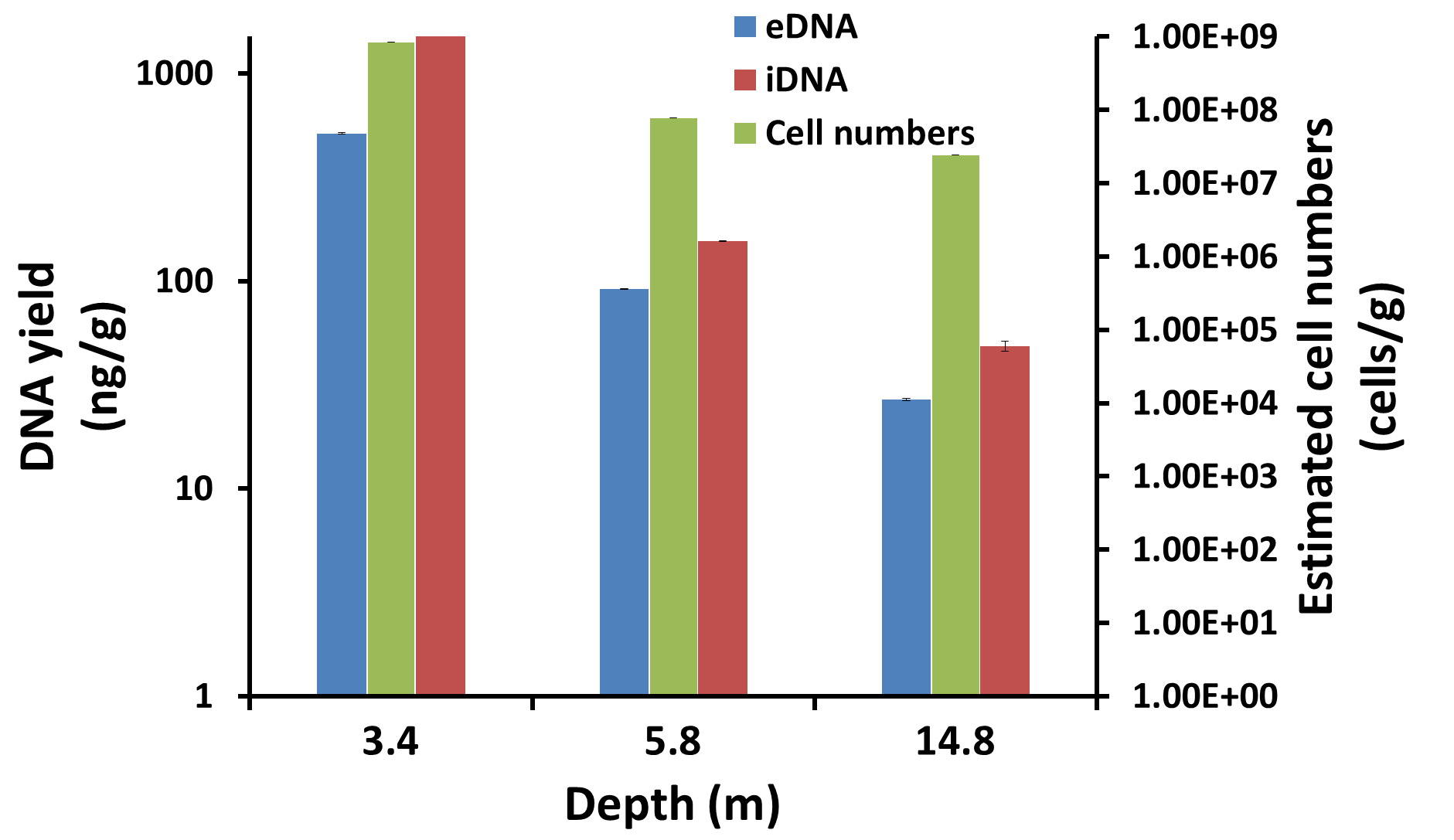


**Figure S5** Yield of iDNA and eDNA fractions from ancient permafrost sediment at 3.4, 5.8 and 14.8 m. The green bars represent the estimated cell numbers from the intracellular DNA fraction by assuming 2×10^-15^ g DNA/cell.


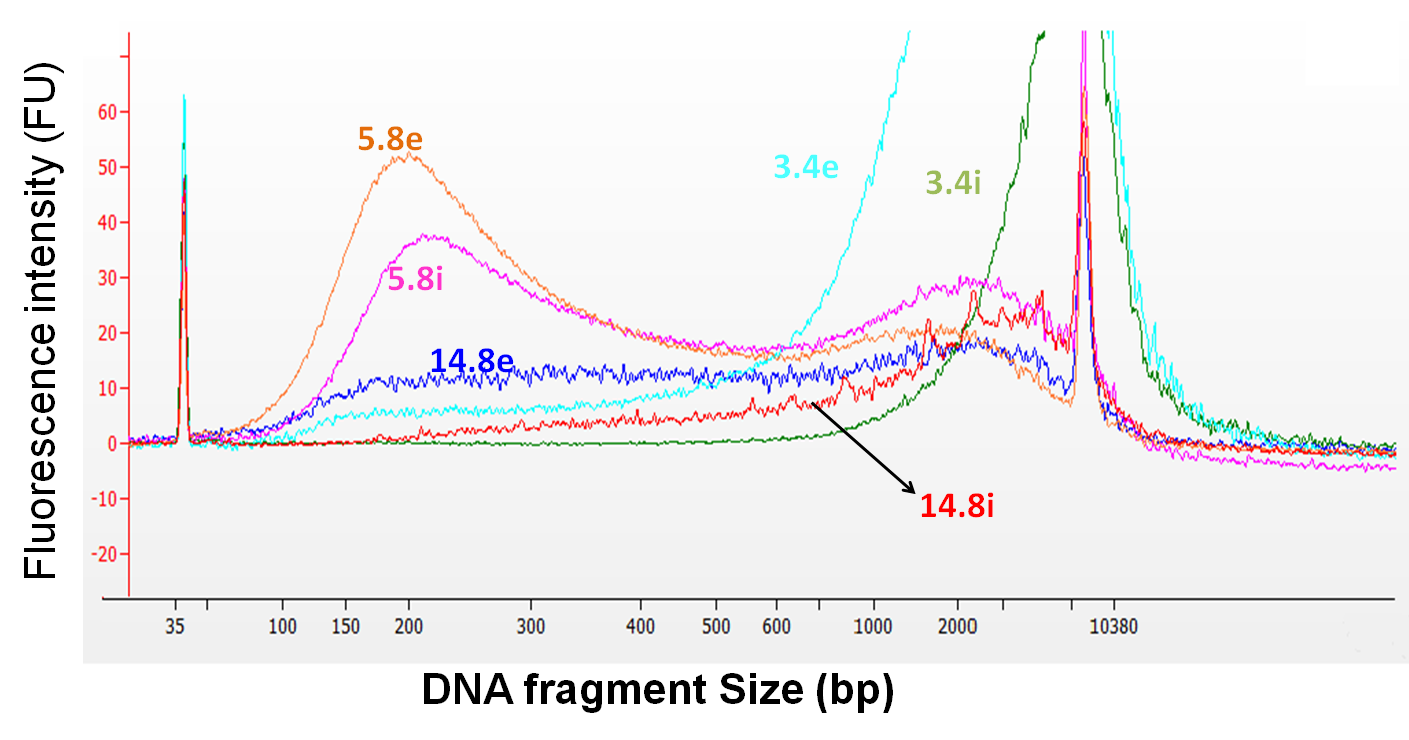


**Figure S6** Size distribution of DNA fragments in iDNA (3.4i, 5.8i and 14.8i) and eDNA (3.4e, 5.8e and 14.8e) fractions from ancient permafrost sediment at 3.4, 5.8 and 14.8 m. The top peaks at 10380 bp for 3.4iDNA and 3.4eDNA sampels were cropped out due to the much higher concentration.


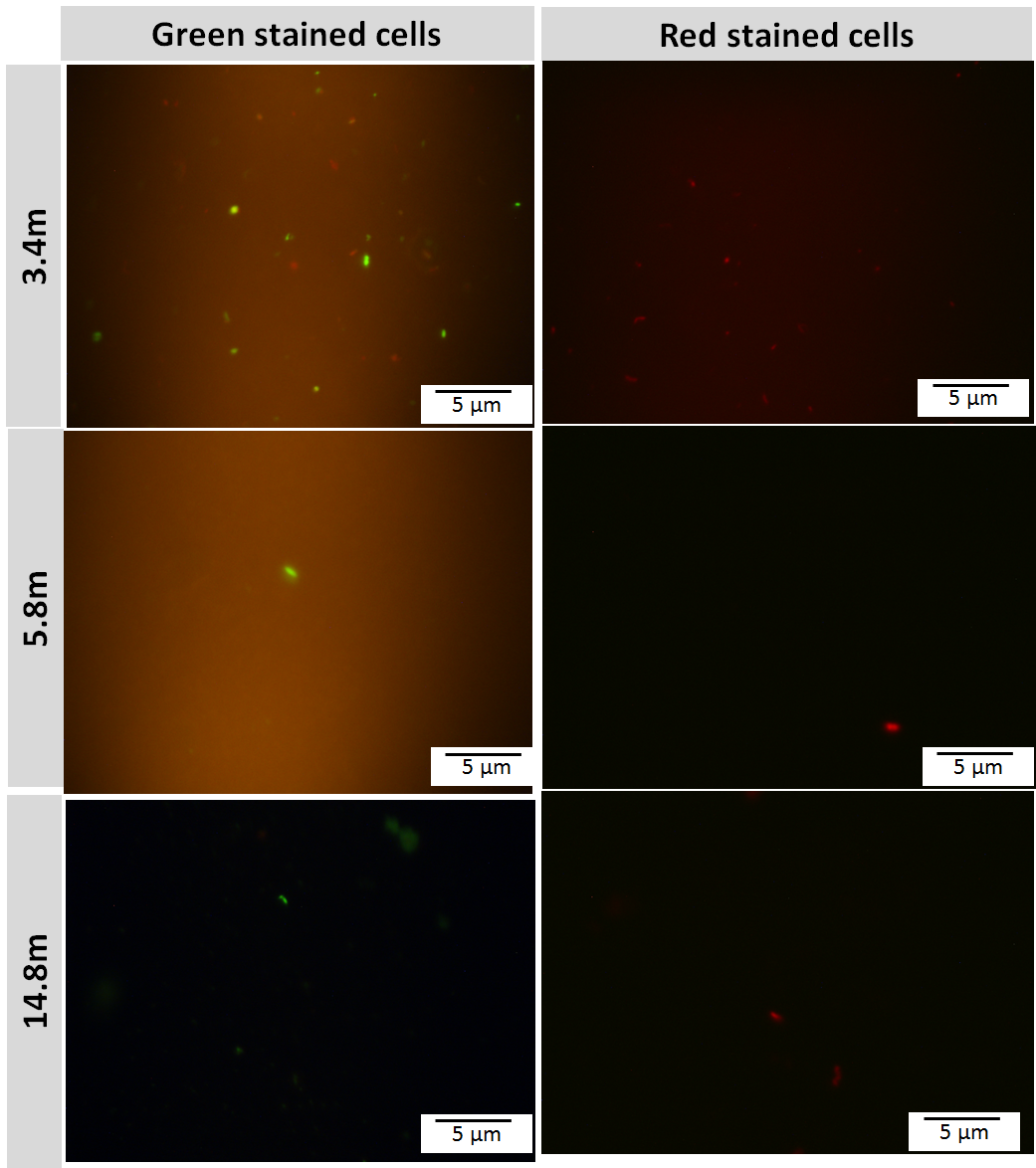


**Figure S7** Live/Dead cell staining of separated cells from ancient permafrost sediments at 3.4 (top), 5.8 (middle) and 14.8 m (bottom). The green stained live cells by Syto9 are shown in the left panel whereas the red stained dead cells are depicted in the right panel.


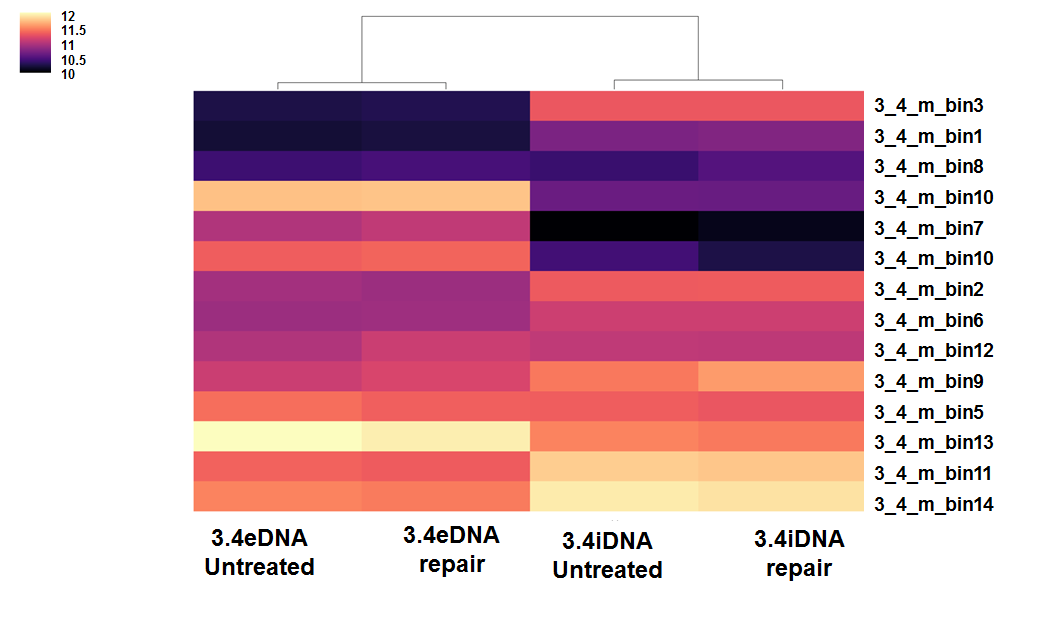


**Figure S8** Relative abundance of MAGs from each metagenome of iDNA and eDNA at 3.4 m with and without PreCR DNA repair. The scale bar indicates the relative abundance of each MAG normalized to the individual sample size as genome copies per million reads.


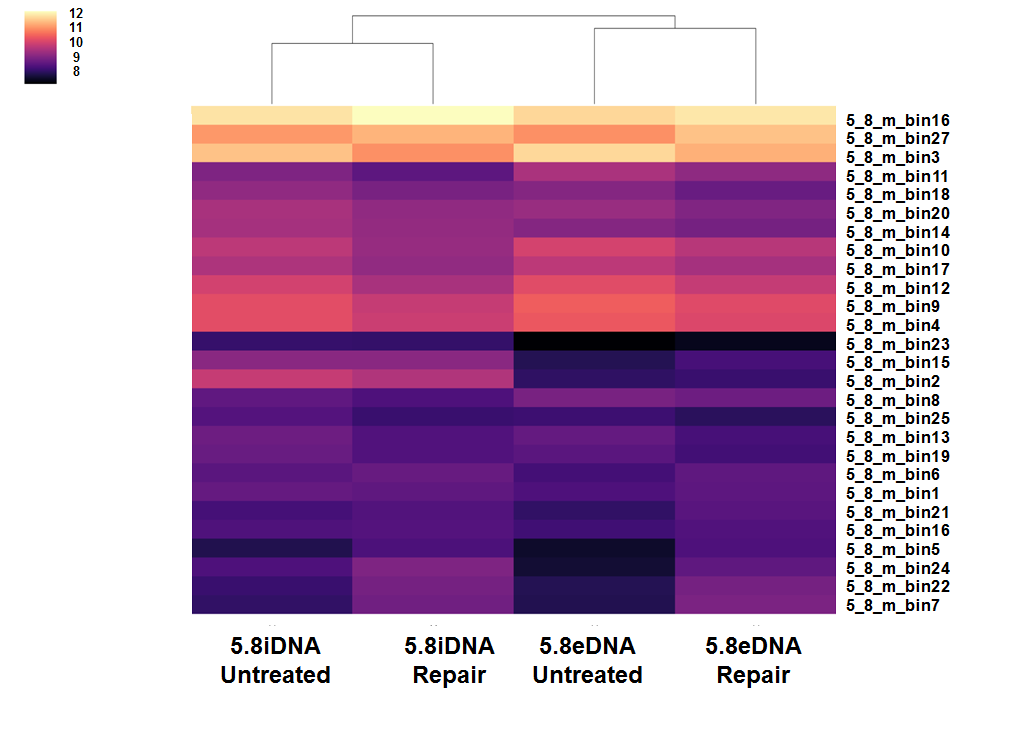


**Figure S9** Relative abundance of MAGs from each metagenome of iDNA and eDNA at 5.8 m with and without PreCR DNA repair. The scale bar indicates the relative abundance of each MAG normalized to the individual sample size as genome copies per million reads.


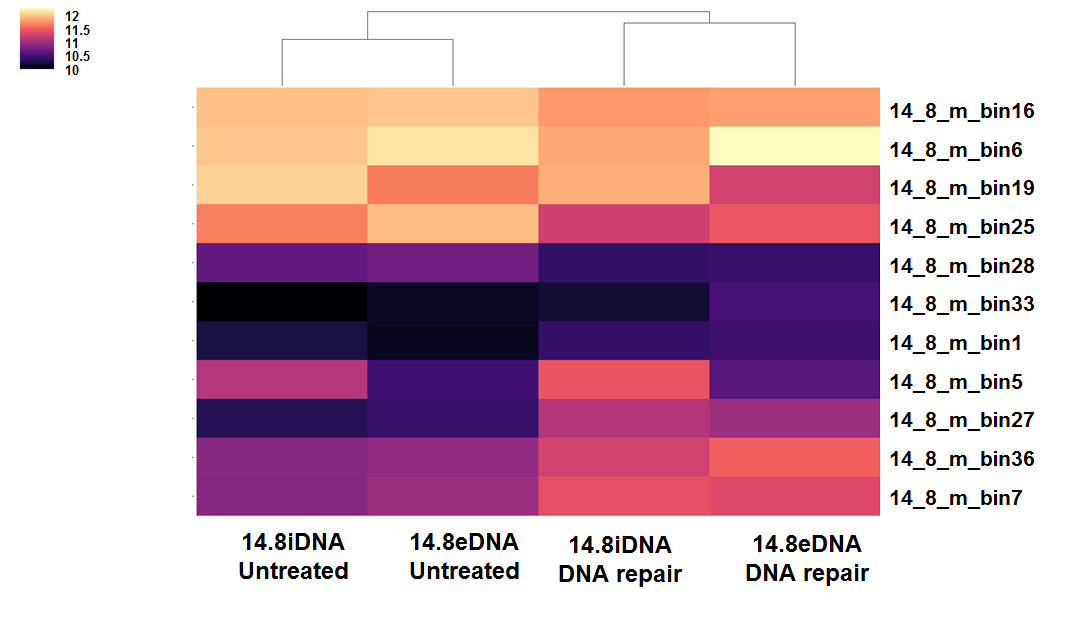


**Figure S10** Relative abundance of MAGs from each metagenome of iDNA and eDNA at 14.8 m with and without PreCR DNA repair. The scale bar indicates the relative abundance of each MAG normalized to the individual sample size as genome copies per million reads.


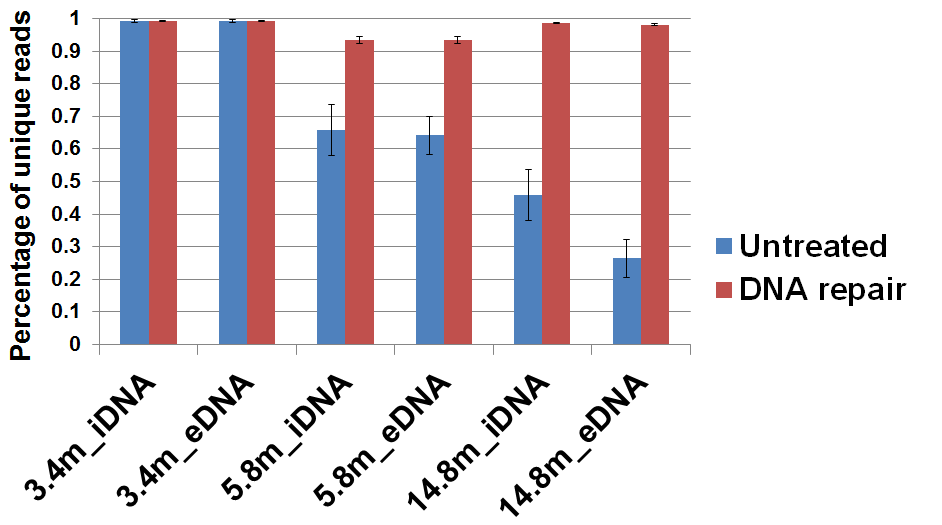


**Figure S11** Percentage of unique reads in each metagenome generated from iDNA and eDNA fractions of the 3.4, 5.8 and 14.8 m samples with and without PreCR DNA repair.


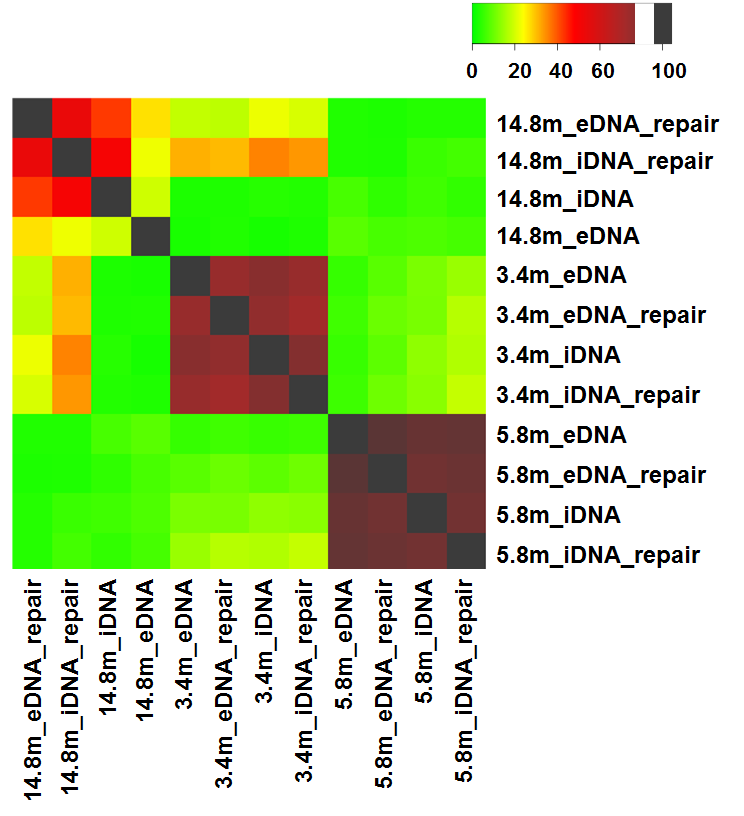


**Figure S12** Heatmap of the global similarity of each iDNA and eDNA derived metagenomes from the 3.4, 5.8 and 14.8 m samples with and without PreCR DNA repair. The similarity matrix was calculated from the similarity of reads in each metagenome. The scale bar represents the normalized percentage of similarity between two metagenomes with respect to the total number of reads in each metagenome.


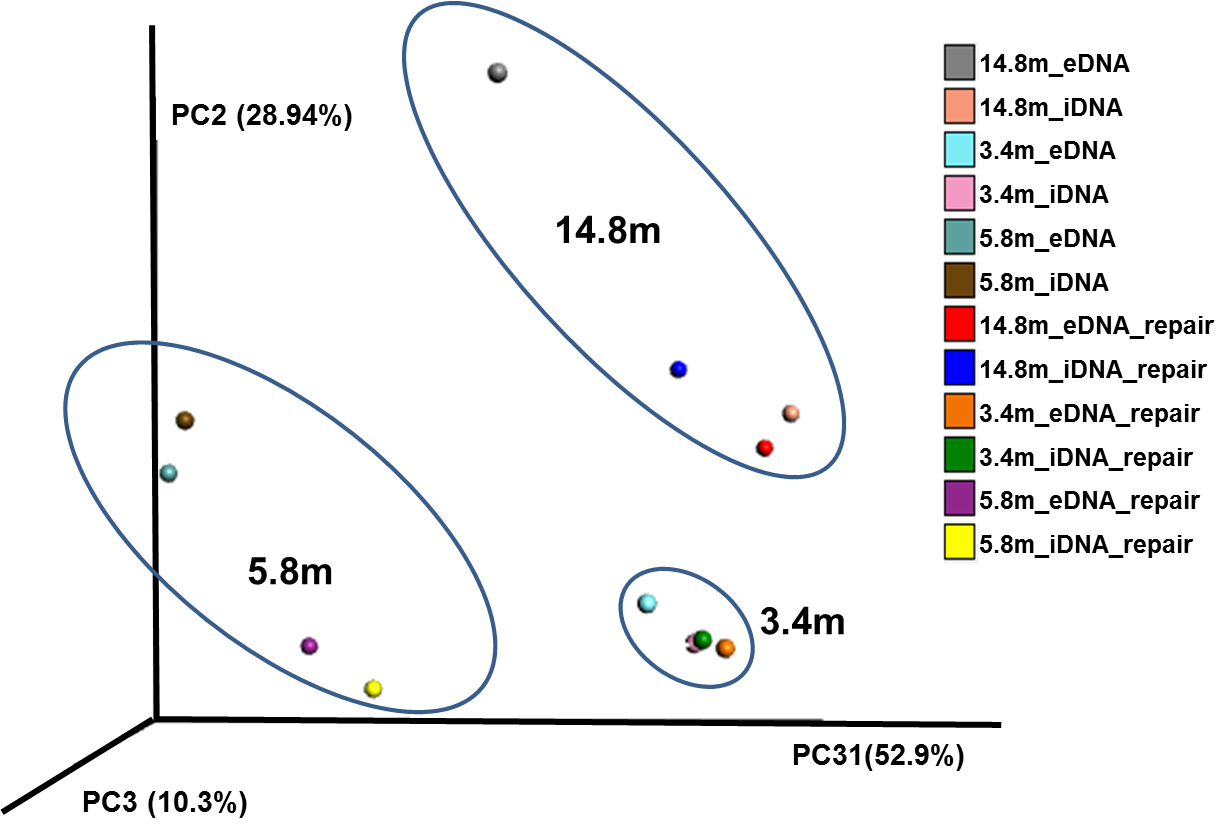


**Figure S13** Principal coordinate analyses (PCoA) of weighted UniFrac distances derived from the microbial community based on the 16S rRNA genes retrieved from each metagenomic dataset of iDNA and eDNA fraction with and without PreCR DNA repair.


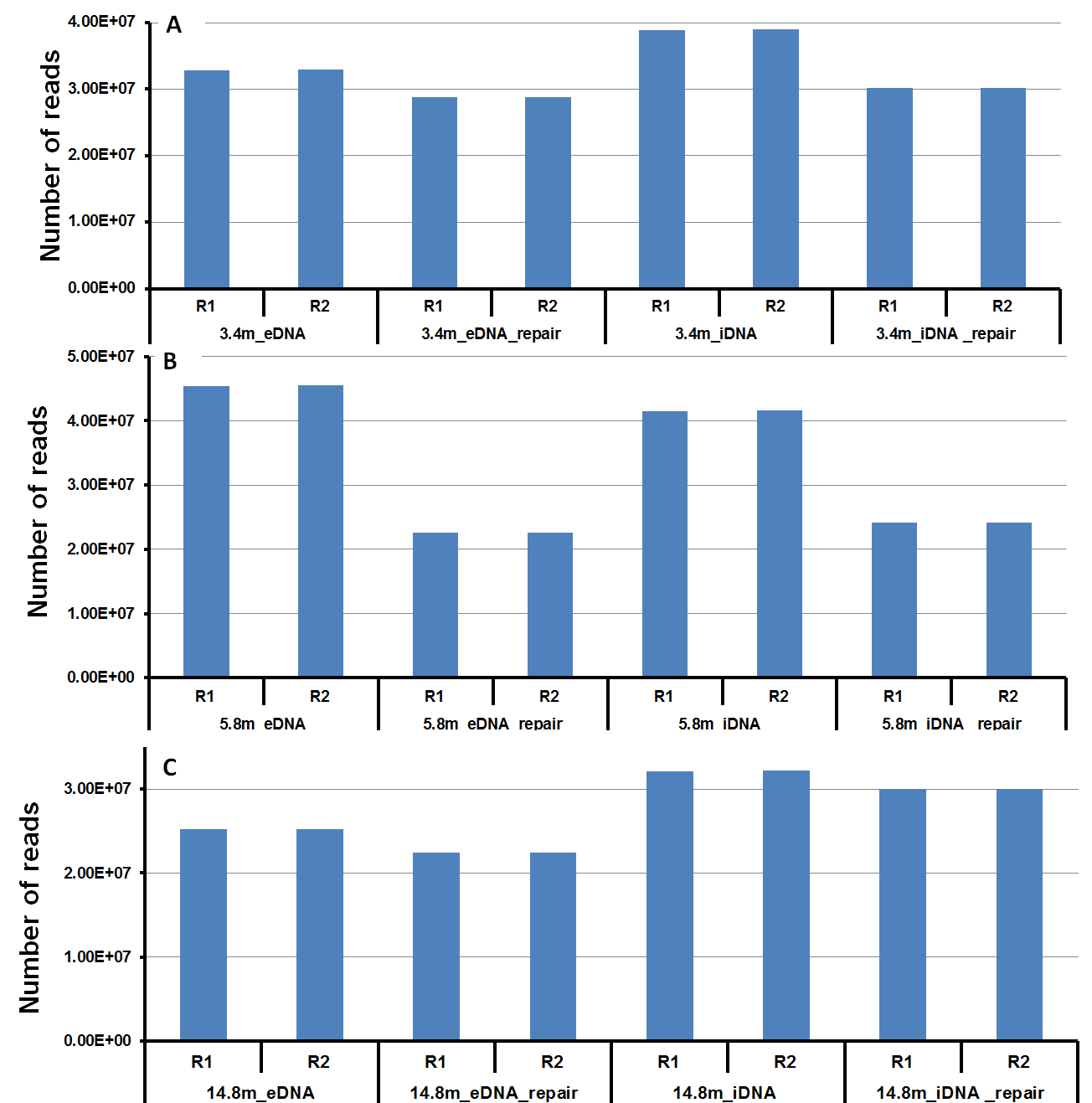


**Figure S14** Number of quality-filtered reads in each metagenome generated from iDNA and eDNA fractions extracted from ancient permafrost sediment at 3.4, 5.8 and 14.8 m with and without PreCR DNA repair.

**
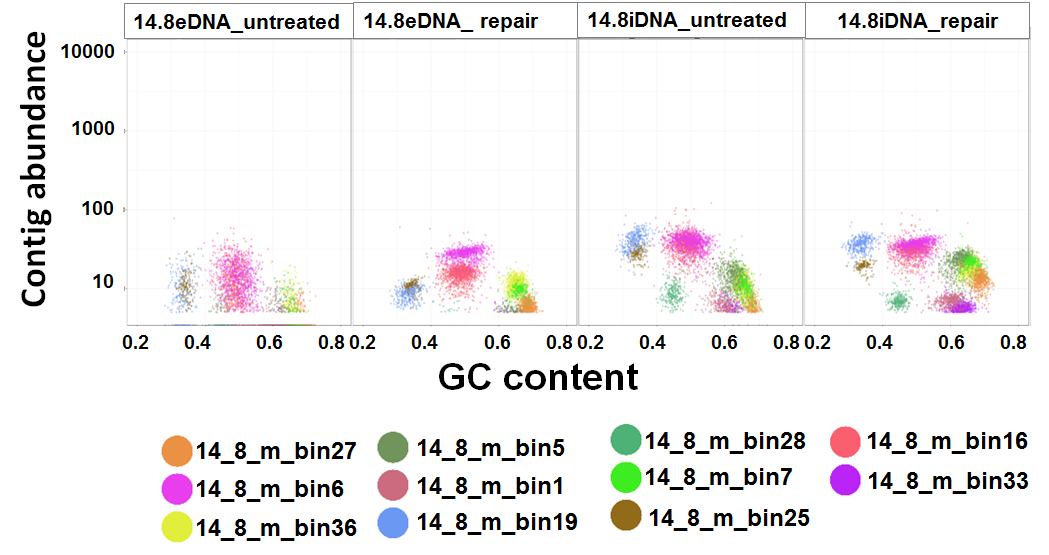
**

**Figure S15** Plot of GC content and contig abundance in each MAG recovered from individual metagenomes of the iDNA and eDNA extracted from the 14.8 m with and without PreCR DNA repair.


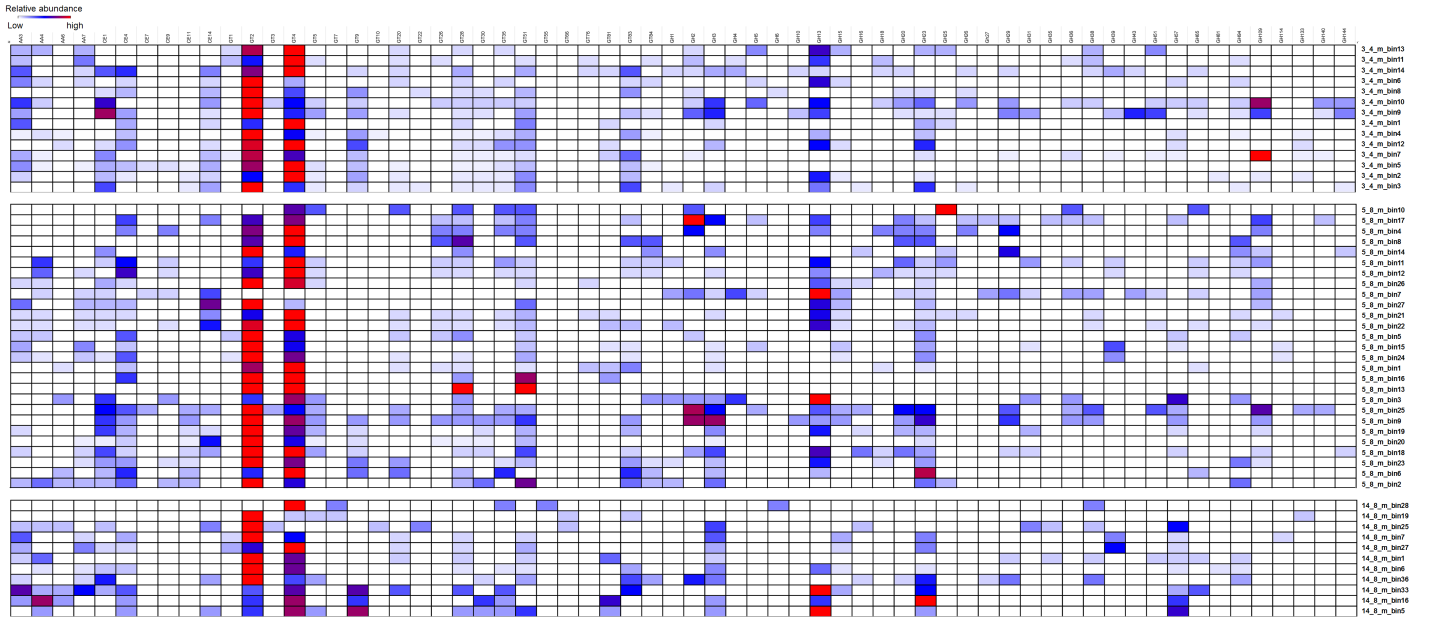


**Figure S16** Groups of carbohydrates active enzymes identified in MAGs recovered from three depths at 3.4, 5.8 and 14.8 m, respectively. The abbreviations for the enzymes classes are as follow: The glycoside hydrolases (GHs), glycosyl transferases (GTs), carbohydrate esterases (CEs) and auxiliary activities (AAs). The relative abundance represents the number of carbohydrates active enzymes identified in each specific subgroup.


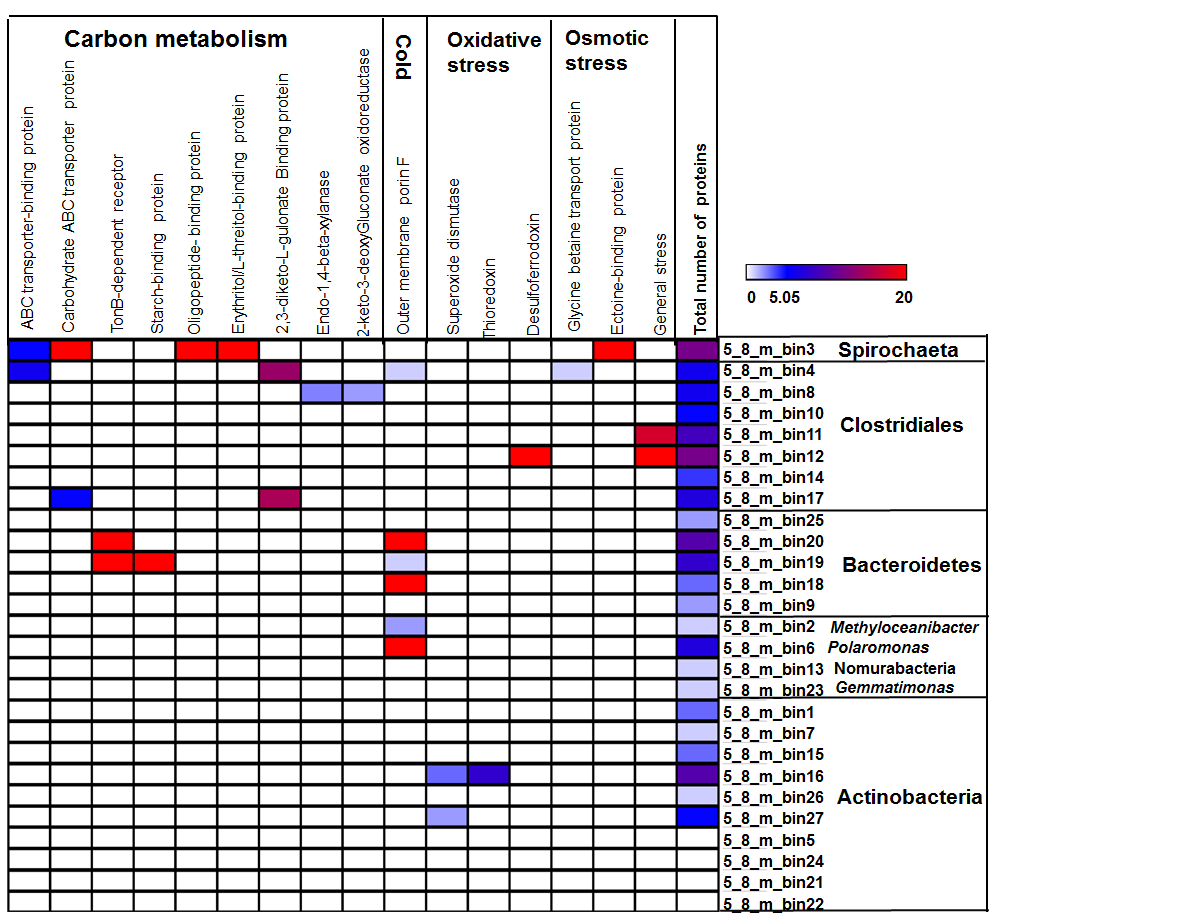


**Figure S17** Relative abundance of proteins identified in the metaproteome from each MAG recovered from the 5.8 m sample. These proteins were involved in carbon and energy metabolism and coping with cold, osmotic and oxidative stresses. The scale bar indicates the balanced spectral counts of proteins.


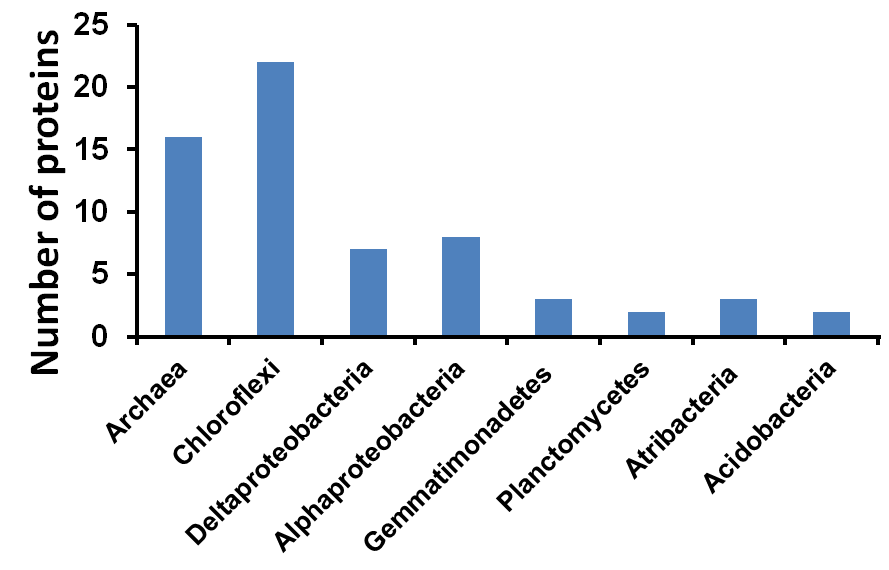


**Figure S18** Number of identified proteins in the metaproteomic dataset at 14.8 m when all genes from the metagenome were used as database for search.

**
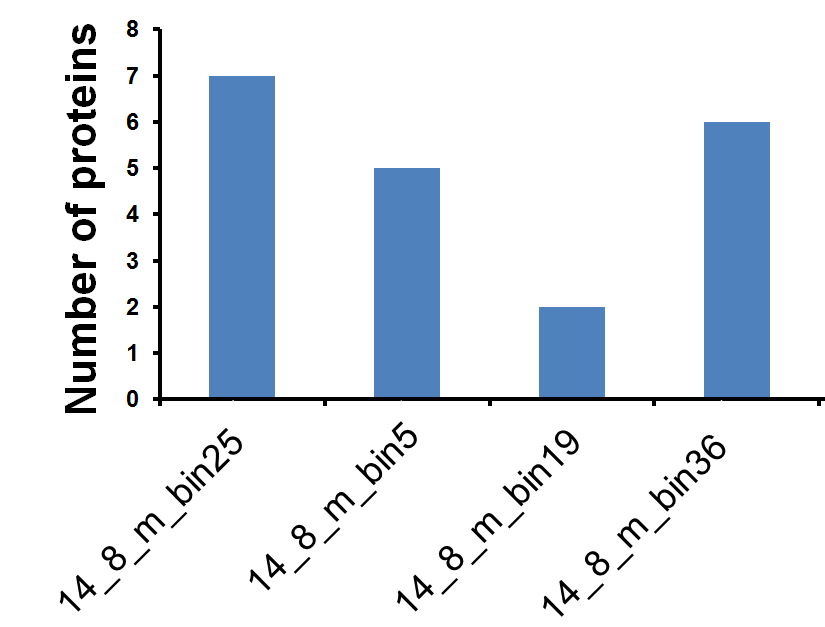
**

**Figure S19** Number of identified proteins from each MAG recovered from 14.8m.


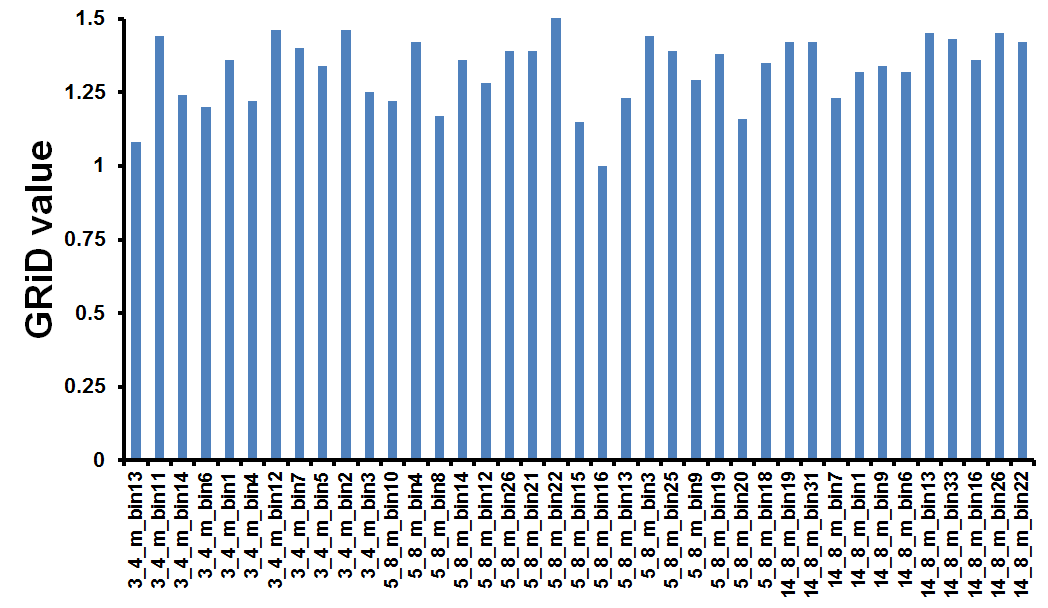


**Figure S20** GRiD measurement of bacterial MAGs from the iDNA metagenomic datasets from ancient permafrost sediment at 3.4, 5.8 and 14.8m. The criterion of valid GRiD values (dnaA/ori and ter/dif ratios > 0.8) for each MAGs was selected according to the output results from the GRiD tool.
