# Supplementary material for: Genomic reconstruction of fossil and living microorganisms in ancient Siberian permafrost
Source: Microbiome. 2021 May 17;9:110. doi: 10.1186/s40168-021-01057-2 (PMC8130349; doi:10.1186/s40168-021-01057-2)
Supplement: Supplementary file 3 — Additional file 2: Table S1. Statistical summary for MAGs recovered from permafrost sediment samples at 3.4, 5.8 and 14.8 m. Note: CDS refers to protein coding sequence. [file 40168_2021_1057_MOESM2_ESM.docx]

**Table S1** Statistical summary for MAGs recovered from permafrost sediment samples at 3.4, 5.8 and 14.8 m. Note: CDS refers to protein coding sequence.

| MAGs | Completeness (%) | Contamination (%) | GC (%) | Number of CDS | N50  (kb) | Size (Mb) | Number of scaffolds |
| --- | --- | --- | --- | --- | --- | --- | --- |
| 3_4_m_bin1 | 86.33 | 2.29 | 58.5 | 2184 | 8.13 | 2.06 | 324 |
| 3_4_m_bin2 | 83.04 | 1.71 | 63.8 | 3527 | 9.25 | 3.85 | 535 |
| 3_4_m_bin3 | 85.99 | 3.42 | 62.7 | 2936 | 17.92 | 3.19 | 247 |
| 3_4_m_bin4 | 94.15 | 0.75 | 53.9 | 3846 | 47.78 | 4.907 | 123 |
| 3_4_m_bin5 | 85.28 | 5.66 | 67.5 | 3928 | 13.02 | 4.26 | 448 |
| 3_4_m_bin6 | 97.44 | 2.14 | 60.5 | 3590 | 17.19 | 3.49 | 341 |
| 3_4_m_bin7 | 93.08 | 3.51 | 60.2 | 5475 | 13.5 | 6.08 | 562 |
| 3_4_m_bin8 | 92.38 | 3.13 | 41.1 | 2650 | 5.7 | 3.08 | 853 |
| 3_4_m_bin9 | 89.67 | 3.25 | 38.1 | 3165 | 9.94 | 3.76 | 725 |
| 3_4_m_bin10 | 82.53 | 2.94 | 40.4 | 2977 | 11.02 | 3.41 | 528 |
| 3_4_m_bin11 | 84.37 | 3.37 | 63.9 | 3887 | 7.33 | 3.76 | 621 |
| 3_4_m_bin12 | 93.18 | 6.36 | 56.7 | 3495 | 15.48 | 3.35 | 284 |
| 3_4_m_bin13 | 93.52 | 1.08 | 70.9 | 3283 | 119.32 | 3.44 | 50 |
| 3_4_m_bin14 | 90.31 | 3.92 | 67.7 | 3092 | 8.13 | 3.06 | 499 |
| 5_8_m_bin1 | 87.93 | 1.58 | 57.8 | 1894 | 10.84 | 1.89 | 256 |
| 5_8_m_bin2 | 93.16 | 5.25 | 63.5 | 3618 | 7.78 | 3.11 | 584 |
| 5_8_m_bin3 | 81.13 | 0 | 29.8 | 2003 | 8.82 | 2.39 | 384 |
| 5_8_m_bin4 | 87.61 | 1.93 | 37.5 | 1844 | 5.96 | 2.05 | 545 |
| 5_8_m_bin5 | 80.01 | 2.90 | 66.43 | 1604 | 3.01 | 1.52 | 590 |
| 5_8_m_bin6 | 84.61 | 6.91 | 59.5 | 4186 | 6.6 | 3.88 | 689 |
| 5_8_m_bin7 | 88.19 | 6.45 | 70.7 | 3145 | 6.58 | 3.18 | 585 |
| 5_8_m_bin8 | 98.10 | 1.06 | 41.1 | 1833 | 29.6 | 1.83 | 103 |
| 5_8_m_bin9 | 92.99 | 0.10 | 33.8 | 1554 | 8.19 | 1.85 | 309 |
| 5_8_m_bin10 | 85.10 | 0 | 33.6 | 1536 | 35.45 | 1.63 | 82 |
| 5_8_m_bin11 | 89.03 | 1.55 | 31.4 | 3269 | 9.47 | 3.53 | 448 |
| 5_8_m_bin12 | 92.45 | 4.76 | 31.2 | 3869 | 37.6 | 3.97 | 167 |
| 5_8_m_bin13 | 82.02 | 1.72 | 32.5 | 739 | 21.18 | 0.73 | 56 |
| 5_8_m_bin14 | 96.69 | 1.45 | 39.5 | 2101 | 19.35 | 2.23 | 217 |
| 5_8_m_bin15 | 98.7 | 0 | 64.2 | 2701 | 133.5 | 2.82 | 41 |
| 5_8_m_bin16 | 94.58 | 0.83 | 52.6 | 1326 | 86.65 | 1.41 | 24 |
| 5_8_m_bin17 | 81.39 | 3.56 | 36.7 | 2853 | 5.95 | 2.64 | 562 |
| 5_8_m_bin18 | 83.30 | 5.52 | 33.4 | 2800 | 16.32 | 3.12 | 283 |
| 5_8_m_bin19 | 87.89 | 3.71 | 37.9 | 2809 | 10.69 | 2.95 | 431 |
| 5_8_m_bin20 | 96.65 | 1.23 | 39.2 | 3256 | 128.6 | 3.39 | 71 |
| 5_8_m_bin21 | 93.55 | 2.98 | 64.7 | 2745 | 11.3 | 2.49 | 284 |
| 5_8_m_bin22 | 97.01 | 2.13 | 69.8 | 3768 | 10.94 | 3.65 | 173 |
| 5_8_m_bin23 | 83.37 | 2.19 | 67.3 | 3048 | 3.85 | 2.91 | 916 |
| 5_8_m_bin24 | 93.96 | 3.01 | 68.9 | 3023 | 21.09 | 2.94 | 213 |
| 5_8_m_bin25 | 93.64 | 0.63 | 46.2 | 2103 | 7.92 | 2.44 | 436 |
| 5_8_m_bin26 | 87.61 | 5.67 | 58.4 | 2055 | 4.83 | 1.87 | 467 |
| 5_8_m_bin27 | 81.44 | 0.59 | 63.2 | 2125 | 3.09 | 1.87 | 718 |
| 14_8_m_bin1 | 89.31 | 1.70 | 59.3 | 2019 | 7.26 | 2.07 | 393 |
| 14_8_m_bin5 | 90.82 | 0.04 | 62.5 | 1918 | 3.63 | 1.88 | 580 |
| 14_8_m_bin6 | 80.45 | 6.36 | 49.7 | 2906 | 5.16 | 3.2 | 759 |
| 14_8_m_bin7 | 94.19 | 1.14 | 66.01 | 2203 | 16.53 | 2.25 | 238 |
| 14_8_m_bin16 | 87.58 | 2.92 | 48.6 | 2984 | 4.17 | 3.08 | 1121 |
| 14_8_m_bin19 | 85.11 | 4.67 | 33.9 | 1329 | 5.59 | 1.26 | 408 |
| 14_8_m_bin25 | 94.39 | 8.87 | 34.2 | 3233 | 47.3 | 3.49 | 141 |
| 14_8_m_bin27 | 86.73 | 8.39 | 68.4 | 2686 | 3.58 | 2.52 | 816 |
| 14_8_m_bin28 | 82.02 | 0.21 | 44.8 | 1578 | 7.37 | 1.46 | 282 |
| 14_8_m_bin33 | 81.57 | 3.42 | 62.9 | 3596 | 5.23 | 3.67 | 864 |
| 14_8_m_bin36 | 89.46 | 1.74 | 64.9 | 3060 | 6.22 | 3.25 | 658 |
