# Supplementary material for: Genomic reconstruction of fossil and living microorganisms in ancient Siberian permafrost
Source: Microbiome. 2021 May 17;9:110. doi: 10.1186/s40168-021-01057-2 (PMC8130349; doi:10.1186/s40168-021-01057-2)
Supplement: Supplementary file 7 — Additional file 6: Table S5. Summary of identified proteins from ancient permafrost at 3.4, 5.8 and 14.8m. [file 40168_2021_1057_MOESM6_ESM.docx]

**Table S5** Summary of identified proteins from ancient permafrost at 3.4, 5.8 and 14.8m.

| **Depths** | **Spectral Counts** | **Peptide counts** | **Protein counts** |
| --- | --- | --- | --- |
| 3.4m | 13422 | 7819 | 2282 |
| 5.8m | 5942 | 1032 | 192 |
| 14.8m | 397 | 222 | 66 |
