# Supplementary material for: Genomic reconstruction of fossil and living microorganisms in ancient Siberian permafrost
Source: Microbiome. 2021 May 17;9:110. doi: 10.1186/s40168-021-01057-2 (PMC8130349; doi:10.1186/s40168-021-01057-2)
Supplement: Supplementary file 10 — Additional file 9. The scripts used for the analyses in this study. [file 40168_2021_1057_MOESM9_ESM.pdf]

Scripts used for

"Genomic reconstruction of fossil and living microorganisms in ancient Siberian permafrost"

Renxing Liang[1*], Zhou Li[2], Maggie C.Y. Lau Vetter[1#], Tatiana A. Vishnivetskaya[3,4], Oksana G. Zanina[4], Karen G. Lloyd[3], Susan Pfiffner[3], Elizaveta M. Rivkina[4], Wei Wang[5], Jessica Wiggins[5], Jennifer Miller[5,] Robert L Hettich[2], Tullis C. Onstott[1]

1. **Quality-control of raw reads of 12 metagenomes (14.8iDNA, 14.8eDNA, 14.8iDNA_preCR, 14.8eDNA_preCR, 14.8iDNA, 14.8eDNA, 14.8iDNA_preCR, 14.8eDNA_preCR, 14.8iDNA, 14.8eDNA, 14.8iDNA_preCR and 14.8eDNA_preCR) with fastp v.0.12.6**

   # fastp -i 3.4i _R1.fastq.gz -I 3.4i_ R2.fastq.gz -o 3.4i_ out.R1.fastq.gz -O 3.4i_ out.R2.fastq.gz -f 1 -t 3 -F 1 -T 3 -l 50 -q 30 -w 12

   # fastp -i 3.4e_ R1.fastq.gz -I 3.4e_ R2.fastq.gz -o 3.4e _out.R1.fastq.gz -O 3.4e_ out.R2.fastq.gz -f 1 -t 3 -F 1 -T 3 -l 50 -q 30 -w 12

   # fastp -i 3.4i_PreCR_R1.fastq.gz -I 3.4i_PreCR_R2.fastq.gz -o 3.4i_PreCR_out.R1.fastq.gz -O 3.4i_PreCR_out.R2.fastq.gz -f 1 -t 3 -F 1 -T 3 -l 50 -q 30 -w 12

   # fastp -i 3.4e_PreCR_R1.fastq.gz -I 3.4e_PreCR_R2.fastq.gz -o 3.4e_PreCR_out.R1.fastq.gz -O 3.4e_PreCR_out.R2.fastq.gz -f 1 -t 3 -F 1 -T 3 -l 50 -q 30 -w 12

   # fastp -i 5.8i _R1.fastq.gz -I 5.8i_ R2.fastq.gz -o 5.8i_ out.R1.fastq.gz -O 5.8i_ out.R2.fastq.gz -f 1 -t 3 -F 1 -T 3 -l 50 -q 30 -w 12

   # fastp -i 5.8e_ R1.fastq.gz -I 5.8e_ R2.fastq.gz -o 5.8e _out.R1.fastq.gz -O 5.8e_ out.R2.fastq.gz -f 1 -t 3 -F 1 -T 3 -l 50 -q 30 -w 12

   # fastp -i 5.8i_PreCR_R1.fastq.gz -I 5.8i_PreCR_R2.fastq.gz -o 5.8i_PreCR_out.R1.fastq.gz -O 5.8i_PreCR_out.R2.fastq.gz -f 1 -t 3 -F 1 -T 3 -l 50 -q 30 -w 12

   # fastp -i 5.8e_PreCR_R1.fastq.gz -I 5.8e_PreCR_R2.fastq.gz -o 5.8e_PreCR_out.R1.fastq.gz -O 5.8e_PreCR_out.R2.fastq.gz -f 1 -t 3 -F 1 -T 3 -l 50 -q 30 -w 12

   # fastp -i 14.8i _R1.fastq.gz -I 14.8i_ R2.fastq.gz -o 14.8i_ out.R1.fastq.gz -O 14.8i_ out.R2.fastq.gz -f 1 -t 3 -F 1 -T 3 -l 50 -q 30 -w 12

   # fastp -i 14.8e_ R1.fastq.gz -I 14.8e_ R2.fastq.gz -o 14.8e _out.R1.fastq.gz -O 14.8e_ out.R2.fastq.gz -f 1 -t 3 -F 1 -T 3 -l 50 -q 30 -w 12

fastp -i 14.8i_PreCR_R1.fastq.gz -I 14.8i_PreCR_R2.fastq.gz -o 14.8i_PreCR_out.R1.fastq.gz -O 14.8i_PreCR_out.R2.fastq.gz -f 1 -t 3 -F 1 -T 3 -l 50 -q 30 -w 12

fastp -i 14.8e_PreCR_R1.fastq.gz -I 14.8e_PreCR_R2.fastq.gz -o 14.8e_PreCR_out.R1.fastq.gz -O 14.8e_PreCR_out.R2.fastq.gz -f 1 -t 3 -F 1 -T 3 -l 50 -q 30 -w 12

2. **Assembly of metagenomic reads with MEGAHIT v1.1.4**

*****Concatenate pair-end reads from metagenomes from 3.4m, 5.8 and 14.8m******

#cat 3.4e_PreCR_out.R1.fastq 3.4e _out.R1.fastq 3.4i_PreCR_R1.fastq 3.4e_PreCR_R1.fastq > 3.4_ALL_READS_1.fastq

#cat 3.4e_PreCR_out.R2.fastq 3.4e _out.R2.fastq 3.4i_PreCR_R2.fastq 3.4e_PreCR_R2.fastq > 3.4_ALL_READS_2.fastq

#cat 5.8e_PreCR_out.R1.fastq 5.8e _out.R1.fastq 5.8i_PreCR_R1.fastq 5.8e_PreCR_R1.fastq > 5.8_ALL_READS_1.fastq

#cat 5.8e_PreCR_out.R2.fastq 5.8e _out.R2.fastq 5.8i_PreCR_R2.fastq 5.8e_PreCR_R2.fastq > 5.8_ALL_READS_2.fastq

#cat 14.8e_PreCR_out.R1.fastq 14.8e _out.R1.fastq 14.8i_PreCR_R1.fastq 14.8e_PreCR_R1.fastq > 14.8_ALL_READS_1.fastq

#cat 14.8e_PreCR_out.R2.fastq 14.8e _out.R2.fastq 14.8i_PreCR_R2.fastq 14.8e_PreCR_R2.fastq > 14.8_ALL_READS_2.fastq

*****Assemble the concatenated reads from 3.4m, 5.8 and 14.8m******

#megahit -1 CLEAN_READS/3.4_ALL_READS_1.fastq -2 CLEAN_READS/3.4_ALL_READS_2.fastq --k-min 27 --k-max 127 --kmin-1pass -m 0.95 -t 32 -o ASSEMBLY

#megahit -1 CLEAN_READS/5.8_ALL_READS_1.fastq -2 CLEAN_READS/5.8_ALL_READS_2.fastq --k-min 27 --k-max 127 --kmin-1pass -m 0.95 -t 32 -o ASSEMBLY

#megahit -1 CLEAN_READS/14.8_ALL_READS_1.fastq -2 CLEAN_READS/14.8_ALL_READS_2.fastq --k-min 27 --k-max 127 --kmin-1pass -m 0.95 -t 32 -o ASSEMBLY

3. **Initial binning from contigs using MetaWRAP v0.8**
   # metawrap binning -o INITIAL_BINNING -t 20 -a ASSEMBLY/megahit/final.contigs.fa --metabat2 --maxbin2 --concoct --run-checkm ../../preCR_3.4i/3_4i_PreCR_out_1.fastq ../../preCR_3.4i/3_4i_PreCR_out_2.fastq ../../preCR_3.4e/3_4e_PreCR_out_1.fastq ../../preCR_3.4e/3_4e_PreCR_out_2.fastq ../../../Ch1_17_no_repair/3.4i/3_4i_out_1.fastq ../../../Ch1_17_no_repair/3.4i/3_4i_out_2.fastq ../../../Ch1_17_no_repair/3.4e/3_4e_out_1.fastq ../../../Ch1_17_no_repair/3.4e/3_4e_out_2.fastq

   # metawrap binning -o INITIAL_BINNING -t 20 -a ASSEMBLY/megahit/final.contigs.fa --metabat2 --maxbin2 --concoct --run-checkm ../../preCR_5.8i/5_8i_PreCR_out_1.fastq ../../preCR_5.8i/5_8i_PreCR_out_2.fastq ../../preCR_5.8e/5_8e_PreCR_out_1.fastq ../../preCR_5.8e/5_8e_PreCR_out_2.fastq ../../../Ch1_17_no_repair/5.8i/5_8i_out_1.fastq ../../../Ch1_17_no_repair/5.8i/5_8i_out_2.fastq ../../../Ch1_17_no_repair/5.8e/5_8e_out_1.fastq ../../../Ch1_17_no_repair/5.8e/5_8e_out_2.fastq

   # metawrap binning -o INITIAL_BINNING -t 20 -a ASSEMBLY/megahit/final.contigs.fa --metabat2 --maxbin2 --concoct --run-checkm ../../preCR_14.8i/14_8i_PreCR_out_1.fastq ../../preCR_14.8i/14_8i_PreCR_out_2.fastq ../../preCR_14.8e/14_8e_PreCR_out_1.fastq ../../preCR_14.8e/14_8e_PreCR_out_2.fastq ../../../Ch1_17_no_repair/14.8i/14_8i_out_1.fastq ../../../Ch1_17_no_repair/14.8i/14_8i_out_2.fastq ../../../Ch1_17_no_repair/14.8e/14_8e_out_1.fastq ../../../Ch1_17_no_repair/14.8e/14_8e_out_2.fastq

4. **Refinement of MAGs (>50% completeness and < 10% contamination) in in MetaWRAP v0.8**

   # metawrap bin_refinement -o BIN_REFINEMENT_3_4_MAGs_50 -t 20 -A INITIAL_BINNING/metabat2_bins -B INITIAL_BINNING/concoct_bins -C INITIAL_BINNING/maxbin2_bins -c 50 -x 10

   # metawrap bin_refinement -o BIN_REFINEMENT_5_8_MAGs_50 -t 20 -A INITIAL_BINNING/metabat2_bins -B INITIAL_BINNING/concoct_bins -C INITIAL_BINNING/maxbin2_bins -c 50 -x 10

metawrap bin_refinement -o BIN_REFINEMENT_14_8_MAGs_50 -t 20 -A INITIAL_BINNING/metabat2_bins -B INITIAL_BINNING/concoct_bins -C INITIAL_BINNING/maxbin2_bins -c 50 -x 10

5. **Characterization of MAGs using the "blobology" module in MetaWRAP v0.8**
   #metawrap blobology -a ASSEMBLY/megahit/final.contigs.fa -t 28 -o BLOBOLOGY --bins BIN_REFINEMENT_3_4_MAGs_50 /metaWRAP_bins ../../preCR_3.4i/3_4i_PreCR_out_1.fastq ../../preCR_3.4i/3_4i_PreCR_out_2.fastq ../../preCR_3.4e/3_4e_PreCR_out_1.fastq ../../preCR_3.4e/3_4e_PreCR_out_2.fastq ../../../Ch1_17_no_repair/3.4i/3_4i_out_1.fastq ../../../Ch1_17_no_repair/3.4i/3_4i_out_2.fastq ../../../Ch1_17_no_repair/3.4e/3_4e_out_1.fastq ../../../Ch1_17_no_repair/3.4e/3_4e_out_2.fastq

   #metawrap blobology -a ASSEMBLY/megahit/final.contigs.fa -t 28 -o BLOBOLOGY --bins BIN_REFINEMENT_5_8_MAGs_50 /metaWRAP_bins ../../preCR_5.8i/5_8i_PreCR_out_1.fastq ../../preCR_5.8i/5_8i_PreCR_out_2.fastq ../../preCR_5.8e/5_8e_PreCR_out_1.fastq ../../preCR_5.8e/5_8e_PreCR_out_2.fastq ../../../Ch1_17_no_repair/5.8i/5_8i_out_1.fastq ../../../Ch1_17_no_repair/5.8i/5_8i_out_2.fastq ../../../Ch1_17_no_repair/5.8e/5_8e_out_1.fastq ../../../Ch1_17_no_repair/5.8e/5_8e_out_2.fastq

   #metawrap blobology -a ASSEMBLY/megahit/final.contigs.fa -t 28 -o BLOBOLOGY --bins BIN_REFINEMENT_14_8_MAGs_50 /metaWRAP_bins ../../preCR_14.8i/14_8i_PreCR_out_1.fastq ../../preCR_14.8i/14_8i_PreCR_out_2.fastq ../../preCR_14.8e/14_8e_PreCR_out_1.fastq ../../preCR_14.8e/14_8e_PreCR_out_2.fastq ../../../Ch1_17_no_repair/14.8i/14_8i_out_1.fastq ../../../Ch1_17_no_repair/14.8i/14_8i_out_2.fastq ../../../Ch1_17_no_repair/14.8e/14_8e_out_1.fastq ../../../Ch1_17_no_repair/14.8e/14_8e_out_2.fastq

6. **Relative abundance of MAGs using the "quant_bins" module in MetaWRAP v0.8**
   #metawrap quant_bins -b BIN_REFINEMENT_3_4_MAGs_50 /metaWRAP_bins -t 12 -o QUANT_BINS -a ASSEMBLY/megahit/final.contigs.fa ../../preCR_3.4i/3_4i_PreCR_out_1.fastq ../../preCR_3.4i/3_4i_PreCR_out_2.fastq ../../preCR_3.4e/3_4e_PreCR_out_1.fastq ../../preCR_3.4e/3_4e_PreCR_out_2.fastq ../../../Ch1_17_no_repair/3.4i/3_4i_out_1.fastq ../../../Ch1_17_no_repair/3.4i/3_4i_out_2.fastq ../../../Ch1_17_no_repair/3.4e/3_4e_out_1.fastq ../../../Ch1_17_no_repair/3.4e/3_4e_out_2.fastq

#metawrap quant_bins -b BIN_REFINEMENT_5_8_MAGs_50 /metaWRAP_bins -t 12 -o QUANT_BINS -a ASSEMBLY/megahit/final.contigs.fa ../../preCR_5.8i/5_8i_PreCR_out_1.fastq ../../preCR_5.8i/5_8i_PreCR_out_2.fastq ../../preCR_5.8e/5_8e_PreCR_out_1.fastq ../../preCR_5.8e/5_8e_PreCR_out_2.fastq ../../../Ch1_17_no_repair/5.8i/5_8i_out_1.fastq ../../../Ch1_17_no_repair/5.8i/5_8i_out_2.fastq ../../../Ch1_17_no_repair/5.8e/5_8e_out_1.fastq ../../../Ch1_17_no_repair/5.8e/5_8e_out_2.fastq

#metawrap quant_bins -b BIN_REFINEMENT_14_8_MAGs_50 /metaWRAP_bins -t 12 -o QUANT_BINS -a ASSEMBLY/megahit/final.contigs.fa ../../preCR_5.8i/14_8i_PreCR_out_1.fastq ../../preCR_5.8i/14_8i_PreCR_out_2.fastq ../../preCR_5.8e/14_8e_PreCR_out_1.fastq ../../preCR_5.8e/14_8e_PreCR_out_2.fastq ../../../Ch1_17_no_repair/5.8i/14_8i_out_1.fastq ../../../Ch1_17_no_repair/5.8i/14_8i_out_2.fastq ../../../Ch1_17_no_repair/5.8e/14_8e_out_1.fastq ../../../Ch1_17_no_repair/5.8e/14_8e_out_2.fastq

7. **Example of mapping MGAs to each metagenome from iDNA and eDNA fractions without and without DNA repair**

#metawrap reassemble_bins -o BIN__preCR_3.4i -1 ../../../preCR_3.4i/3_4i_PreCR_out_1.fastq -2 ../../../preCR_3.4i/3_4i_PreCR_out_2.fastq -t 28 -m 112 -c 20 -x 50 -b ../BIN_REFINEMENT/metaWRAP_bins

#metawrap reassemble_bins -o BIN_REASSEMBLY_preCR_3.4e -1 ../../../preCR_3.4e/3_4e_PreCR_out_1.fastq -2 ../../../preCR_3.4e/3_4e_PreCR_out_2.fastq -t 28 -m 112 -c 20 -x 50 -b ../BIN_REFINEMENT/metaWRAP_bins

#metawrap reassemble_bins -o BIN_REASSEMBLY_3.4i -1 ../../../../Ch1_17_no_repair/3.4i/3_4i_out_1.fastq -2 ../../../../Ch1_17_no_repair/3.4i/3_4i_out_2.fastq -t 28 -m 112 -c 20 -x 50 -b ../BIN_REFINEMENT/metaWRAP_bins

#metawrap reassemble_bins -o BIN_REASSEMBLY_3.4e -1 ../../../../Ch1_17_no_repair/3.4e/3_4e_out_1.fastq -

2 ../../../../Ch1_17_no_repair/3.4e/3_4e_out_2.fastq -t 28 -m 112 -c 20 -x 50 -b ../BIN_REFINEMENT/metaWRAP_bins

8. Reassembly of MAGs using "Reassemble_bins module" in MetaWRAP v0.8

metawrap reassemble_bins -o 3_4_ reassembled_bins -1 CLEAN_READS/ALL_READS_1.fastq -2 CLEAN_READS/ALL_READS_2.fastq -t 28 -m 112 -c 80 -x 10 -b BIN_REFINEMENT/metaWRAP_bins

metawrap reassemble_bins -o 5_8_ reassembled_bins -1 CLEAN_READS/ALL_READS_1.fastq -2 CLEAN_READS/ALL_READS_2.fastq -t 28 -m 112 -c 80 -x 10 -b BIN_REFINEMENT/metaWRAP_bins

metawrap reassemble_bins -o 14_8_ reassembled_bins -1 CLEAN_READS/ALL_READS_1.fastq -2 CLEAN_READS/ALL_READS_2.fastq -t 28 -m 112 -c 80 -x 10 -b BIN_REFINEMENT/metaWRAP_bins

9. Assess the MAGs quality with CheckM v1.0.11

checkm lineage_wf -f 3_4_reassembled_bins/CheckM.txt -t 20 -x fa 3_4_reassembled_bins/ 3_4_reassembled_bins/SCG

checkm lineage_wf -f 5_8_reassembled_bins/CheckM.txt -t 20 -x fa 5_8_reassembled_bins/ 5_8_reassembled_bins/SCG

checkm lineage_wf -f 14_8_reassembled_bins/CheckM.txt -t 20 -x fa 14_8_reassembled_bins/ 14_8_reassembled_bins/SCG

10. Annotation of MAGs with Prokka v1.13

metaWRAP annotate_bins -o FUNCT_ANNOT_3_4_reassembled_bins -t 12 -b 3_4_reassembled_bins

metaWRAP annotate_bins -o FUNCT_ANNOT_5_8_reassembled_bins -t 12 -b 5_8_reassembled_bins

metaWRAP annotate_bins -o FUNCT_ANNOT_14_8_reassembled_bins -t 12 -b 14_8_reassembled_bins

11. Example of genome annotation of MAGs with Blastp

```
# blastp -query 3_4_m_bin2.faa -db nr -num_threads 20 -max_target_seqs 10 -outfmt 11
-out 3_4_m_bin2.faa_best10NR.asn
```

12. **Preliminary taxonomic identification using** GTDB-Tk v 0.3.0

```
# gtdbtk classify_wf --extension fa --cpus 20 --genome_3_4_reassembled_bins
/ --out_dir gtdbtk_output

# gtdbtk classify_wf --extension fa --cpus 20 --genome_5_8_reassembled_bins
/ --out_dir gtdbtk_output

# gtdbtk classify_wf --extension fa --cpus 20 --genome_14_8_reassembled_bins
/ --out_dir gtdbtk_output
```

13. **DNA damage assay with mapDamage v2.0**

```
**** example of assessing DNA damage using MAG (i.e., bin5.fasta)*****
#bwa index -a is bin6.fasta

#java -jar /tigress/rliang/tools/picard/build/libs/picard.jar CreateSequenceDictionary
R=bin6.fasta O=bin6.dict

#samtools faidx bin6.fasta
#bwa aln bin6.fasta ../../../../Ch1_17_no_repair/3.4e/3_4e_out_tirm_1.fastq -n 0.1 -l 1000 >
bin6_14.8e.sai
#bwa samse  bin6.fasta
#bin6_14.8e.sai ../../../../Ch1_17_no_repair/3.4e/3_4e_out_tirm_1.fastq > bin6_14.8e.sam
#samtools view -Sb bin6_14.8e.sam > bin6_14.8e.bam

#samtools sort bin6_14.8e.bam -o bin6_14.8e_sort.bam
#samtools index bin6_14.8e_sort.bam
#java -jar /tigress/rliang/tools/picard/build/libs/picard.jar AddOrReplaceReadGroups
INPUT=bin6_14.8e_sort.bam OUTPUT=bin6_14.8e.RG.bam RGID=rg_id RGLB=lib_id
RGPL=platform RGPU=plat_unit RGSM=sam_id
VALIDATION_STRINGENCY=LENIENT

#samtools index bin6_14.8e.RG.bam

#java -jar /tigress/rliang/tools/picard/build/libs/picard.jar MarkDuplicates
I=bin6_14.8e.RG.bam O=bin6_14.8e.DR.bam M=output_metrics.txt
```

```
REMOVE_DUPLICATES=True VALIDATION_STRINGENCY=LENIENT &> logFile.log

#samtools index bin6_14.8e.DR.bam

#java -jar /tigress/rliang/tools/GATK/GenomeAnalysisTK.jar -T RealignerTargetCreator -R  bin6.fasta -I bin6_14.8e.DR.bam -o targets.intervals

#java -jar /tigress/rliang/tools/GATK/GenomeAnalysisTK.jar -T IndelRealigner -R bin6.fasta -I bin6_14.8e.DR.bam -targetIntervals targets.intervals -o bin6_14.8e.final.bam --filter_bases_not_stored &> logFile.log

#samtools sort bin6_14.8e.final.bam -o bin6_14.8e.final.sort.bam
samtools index bin6_14.8e.final.sort.bam

#mapDamage --length 140 --seq-length 25 -i  samtools index bin6_14.8e.final.sort.bam -r  bin6.fasta --rescale
```

14. Phylogenomic analyses

********Convert all MAGs into .db files using anvio5*********

```
#for i in *fa do
	anvi-script-FASTA-to-contigs-db $i done
```

********* Identifying HMM hits that are common in all MAGs*****

```
# anvi-get-sequences-for-hmm-hits --external-genomes external_genomes.txt --list-hmm-sources
```

***** Obtain the amino acid sequences of these HMM hits of selected ribosomal proteins (Campbell_et_al) as described in Methods, and concatenate them into a single sequence per genome.******

```
#anvi-get-sequences-for-hmm-hits --external-genomes external_genomes.txt -o Campbell_concatenated-proteins.fa --hmm-sources Campbell_et_al --gene-names Campbell_gene-names.txt --return-best-hit --get-aa-sequences –concatenate
```

*****Generate phylogenetic tree with with RAxML v8.1.17*****

```
#raxmlHPC-PTHREADS -f a -x 12345 -p 12345 -N 1000 -m PROTGAMMAILGF -T 20 -s Campbell_concatenated-proteins.phy -n Campbell_concatenated_ribosomalprotein
```

15. Metaproteomic analyses

*** The mass spectrometric RAW files were deposited to the ProteomeXchange Consortium via the PRIDE partner repository (identifier number: PXD022683). The datasets are currently private and will be released to public once published. The datasets can be accessed by the reviewers with the account listed below. ******

#Reviewer account details:

Username: reviewer_pxd022683@ebi.ac.uk
Password: 4ANvrpTM

****Build database base on the proteomes of all MAGs with Sipros Ensemble*****
python /tigress/rliang/tools/Sipros/Scripts/sipros_prepare_protein_database.py -i 14_8_all_contigs.faa -o Decoy_target_14_8_all_contigs.fasta -c SiprosConfig.cfg

****Search database to identify potential expressed proteins*****

#Sipros_OpenMP -o Spe2Pep_directory_14.8_MAGs_total -w FT2_files -c SiprosConfig.cfg

#/tigress/rliang/tools/Sipros/Scripts/runSiprosFiltering.sh -in Spe2Pep_directory_14.8_MAGs_total -o output_MAGs_all_0.01 -c SiprosConfig.cfg
